# Hypercoagulation detected by routine and global laboratory hemostasis assays in patients with infective endocarditis

**DOI:** 10.1371/journal.pone.0261429

**Published:** 2021-12-15

**Authors:** Ekaterina M. Koltsova, Maria A. Sorokina, Alexandra S. Pisaryuk, Nikita M. Povalyaev, Anastasia A. Ignatova, Dmitry M. Polokhov, Elizaveta O. Kotova, Alexander V. Balatskiy, Fazoil I. Ataullakhanov, Mikhail A. Panteleev, Zhanna D. Kobalava, Anna N. Balandina

**Affiliations:** 1 Dmitry Rogachev National Medical Research Center of Pediatric Hematology, Oncology and Immunology, Moscow, Russian Federation; 2 Center for Theoretical Problems of Physicochemical Pharmacology, Moscow, Russian Federation; 3 City Clinical Hospital named after V.V. Vinogradov, Moscow, Russia Federation; 4 Peoples’ Friendship University of Russia (RUDN), Moscow, Russia Federation; 5 Lomonosov Moscow State University, Moscow, Russian Federation; 6 Moscow Institute of Physics and Technology, Dolgoprudny, Russian Federation; Universite de Liege (B34), BELGIUM

## Abstract

**Background:**

Coagulation system is heavily involved into the process of infective endocarditis (IE) vegetation formation and can facilitate further embolization. In this study we aimed to assess the coagulation and platelet state in IE implementing a wide range of standard and global laboratory assays. We also aim to determine whether prothrombotic genetic polymorphisms play any role in embolization and mortality in IE patients.

**Methods:**

37 patients with IE were enrolled into the study. Coagulation was assessed using standard coagulation assays (activated partial thromboplastin time (APTT), prothrombin, fibrinogen, D-dimer concentrations) and integral assays (thromboelastography (TEG) and thrombodynamics (TD)). Platelet functional activity was estimated by flow cytometry. Single nuclear polymorphisms of coagulation system genes were studied.

**Results:**

Fibrinogen concentration and fibrinogen-dependent parameters of TEG and TD were increased in patients indicating systemic inflammation. In majority of patients clot growth rate in thrombodynamics was significantly shifted towards hypercoagulation in consistency with D-dimers elevation. However, in some patients prothrombin, thromboelastography and thrombodynamics were shifted towards hypocoagulation. Resting platelets were characterized by glycoprotein IIb-IIIa activation and degranulation. In patients with fatal IE, we observed a significant decrease in fibrinogen and thrombodynamics. In patients with embolism, we observed a significant decrease in the TEG R parameter. No association of embolism or mortality with genetic polymorphisms was found in our cohort.

**Conclusions:**

Our findings suggest that coagulation in patients with infective endocarditis is characterized by general hypercoagulability and platelet pre-activation. Some patients, however, have hypocoagulant coagulation profile, which presumably can indicate progressing of hypercoagulation into consumption coagulopathy.

## Introduction

Infectious endocarditis (IE) is an infection of the heart inner surface mainly affecting heart valves. Despite the fact that infectious endocarditis is a rather rare disease, with the annual incidence ranging from 3 to 7 per 100 000 person-years, it is currently the fourth most common life-threatening infectious syndrome after sepsis, pneumonia and intra-abdominal abscess [1]. The interplay between microorganisms and hemostasis translates into a complex pathophysiologic sequence whose consequences span from septic complications to embolic events (EE) and account for the never-changing mortality of IE reaching 30% despite introduction of antibiotics [2, 3]. Embolic risk is very high in IE, with embolic events occurring in 20–50% of patients [3].

The main cause of EE in IE is the disruption of vegetation [4]. Vegetations are structures formed on the endocardium of heart valves or heart chambers and consisting of fibrin, platelets and bacteria. They originate from primary endocardial injury, followed by focal adherence of platelets and fibrin and then secondarily infected by microorganisms circulating in the blood [5]. These structures subsequently lose rigidity and are disrupt which may result in thromboembolism of pulmonary or other major arteries.

According to current guidelines, the EE prophylaxis in IE includes early antimicrobial therapy and early surgery. The length of vegetation and the history of embolic episodes are the only criteria that are included in international guidelines for assessing embolic risk (ER) and deciding on preventive valve surgery [1, 3]. Hypercoagulation can affect the vegetation formation process [6]. Severe inflammation activates the clotting cascade through the up-regulation of tissue factor on circulating monocytes, tissue macrophages, etc. [7]. At the same time, inflammation suppresses some of the natural anticoagulant mechanisms (concentrations of protein C, antithrombin III and fibrinolytic proteins are reduced) [8]. All together these changes can result in excess thrombin generation, fibrin deposition, and clotting factor consumption in IE patients predisposing them to thrombosis [9].

It is assumed that fragmentation of valvular vegetations as a result of turbulent blood flow in the heart chambers leads to systemic embolism in patients with IE. However, the results of echocardiographic studies do not unambiguously support the simple embolic hypothesis, according to which vegetation is the only mechanism leading to embolic events in these patients [10]. Thus, it is tempting to hypothesize that an inherited prothrombotic condition may synergize with a hypercoagulation present in patients with IE and thus increase embolic risk [11]. Inherited thrombophilias such as G20210A mutation of the prothrombin FII gene or FV Leiden mutation are well-characterized conditions commonly investigated as potential causes of a range of venous and arterial thrombotic disorders [12]. Other coagulation system polumorphisms such as FXIII V34L polymorphism are less studied, but some studies show their potential for thrombosis risk evaluation [13]. As for the fibrinolysis PAI-1 polymorphism may serve as one of the predisposing factors of VTE [14]. Platelet glycoprotein polymorphisms such as) platelet GP Ia/IIa ITGA2 gene polymorphism (rs1126643), platelet GP IIb/IIIa ITGB3 gene polymorphism (rs5918), platelet GP Ib GP1BA gene polymorphism (rs6065), platelet GPVI GP6 gene polymorphism (rs1613662) were associated with myocardial infarction, coronary disease or VTE [12, 15].

The standard coagulation tests that are available in the clinical laboratory are not sensitive to hypercoagulation due to the peculiarities of their design and reagents [16], and the only platelet parameters that can be obtained from the complete blood count (CBC) are platelet number and mean platelet volume. Currently, hemostasis in patients with infective endocarditis is extremely poorly characterized in terms of laboratory tests of hemostasis. With the appearance of more advanced global tests of hemostasis (such as thromboelastography, thrombodynamics, thrombin generation test), as well as more advanced methods for assessing the platelet state (such as evaluation of platelet functional activity by flow cytometry), the diagnosis of hypercoagulation in IE becomes a more feasible task [16–25]. Finding reliable hypercoagulation markers will help to answer to the question about the place of anticoagulant therapy in the treatment of IE.

In this study we aimed to assess the coagulation and platelet state in patients with IE implementing a wide range of standard and advanced laboratory assays available to date. We also aim to determine whether prothrombotic genetic polymorphisms play any role in embolization and mortality in IE patients.

## Materials and methods

### Study population, blood collection and sample transport

We conducted an observational study in 37 patients with IE admitted to City Clinical Hospital named after V.V. Vinogradov, Moscow, Russia throughout 2017–2020. This study enrolled patients older than 18 years old with definite IE diagnosis according to Duke criteria (either 2 major criteria or 1 major and 3 minor criteria or 5 minor criteria) [3]. For further analysis, all patients with IE were divided into groups based on clinical outcomes in form of embolic events or in-hospital mortality. 7 (19%) patients were injecting drug addicts.

Blood samples were collected at three timepoints: at admission and at 5^th^ and 14^th^ day of hospital stay. Blood samples at admission were collected 3–5 hours after making definite IE diagnosis. Blood samples were collected before the regular anticoagulation, if prescribed, for minimization of its impact on test results.

Blood was drawn into vacuum tubes (Monovette, Sarstedt, Germany) with 106 mM sodium citrate buffer (pH 5.5) at a 9:1 blood:anticoagulant volume ratio. Subsequent blood collection was at 5th and 14th day after diagnosis setup. For analysis of platelet function blood was transported (30 min at room temperature) within 1 hour after sampling. All other clotting assays were performed during first 30 min after sampling without transportation.

Written informed consent was given by all participants. All patients were evaluated and treated in accordance with latest European Society of Cardiology (ESC) clinical guidelines [26]. Instrumental examination included transthoracic and transesophageal echocardiography and chest computer tomography (CT) scan. Abdominal ultrasonography, brain magnetic resonance imaging (MRI), heart angiography were performed to rule out embolic events when suspected.

The control groups for tests consisted of healthy volunteers (N = 53 for routine coagulation tests, platelet function tests and thrombodynamics and N = 13 for thromboelastography (TEG)). Only those volunteers who did not have any respiratory diseases and did not take any medication for at least two weeks prior to analysis were included. The exclusion criteria were: 1) abnormal results of CBC or routine coagulation tests (activated partial thromboplastin time (APTT), prothrombin, thrombin time (TT), fibrinogen concentration), 2) chylous plasma, 3) hemolysis.

The study was conducted in accordance with the Helsinki Declaration. The protocol was approved by Local Ethics Committee City Clinical Hospital named after V.V. Vinogradov.

### Therapy

Antibiotic (AB) treatment was administered per the current guidelines of the European Society of Cardiology [26], with regimens of high-dose intravenous medications known to have bactericidal effects against the microorganisms involved. Antibiotic treatment was considered successful if the recommended duration was achieved, and if patients were afebrile, with negative blood cultures. Empiric antibiotic regimens were chosen for IE without identified microorganisms. For these patients, antibiotic treatment was considered successful after at least four to six weeks of duration, clinical recovery, and based on the opinion of the endocarditis specialist. Antiplatelet and anticoagulant agents were administered only in case of already existing or newly diagnosed concurrent conditions, such as coronary heart disease, cerebrovascular disease, atrial fibrillation and mechanical heart prosthetics or immobilization during the treatment.

### Laboratory assays

#### Routine coagulation tests

Routine coagulation tests and D-dimer concentration were measured using an ACL TOP 700 coagulometer (Instrumentation Laboratory, Bedford, MA, USA) in platelet-poor plasma (PPP), obtained by centrifugation at 1750g for 15min. The following assays were performed: APTT (SynthASil, Instrumentation Laboratory, Bedford, MA, USA), prothrombin (RecombiPlasTin 2G, Instrumentation Laboratory, Bedford, MA, USA), fibrinogen concentration (QFA Thrombin, Instrumentation Laboratory, Bedford, MA, USA), D-dimer (HemosIL D-dimer HS, Instrumentation Laboratory, Bedford, MA, USA).

#### Flow cytometry platelet function analysis

Platelet function was assessed as described in [25, 27] with minor modifications. Whole blood samples were diluted with buffer A (150 mM NaCl, 2.7 mM KCl, 1 mM MgCl2, 0.4 mM NaH2PO4, 20 mM HEPES, 5 mM glucose, 0.5% bovine serum albumin, pH 7.4). Platelets were either left intact or loaded with mepacrine (10 μM) for 30 min at 37 °C. Subsequently, platelets were either left unstimulated or stimulated with a mixture of CRP at 10 μg/μl, SFLLRN at 12.5 μM for 10 min in the presence of 2.5 mM calcium chloride. Both resting and activated samples were incubated with antibodies against CD61, CD42b, CD62P, as well as PAC1 and annexin V for 10 min. Subsequently, they were diluted 10-fold with buffer A containing 2.5mM calcium, and analyzed using Novocyte (Acea Bioscience, San Diego, CA, USA) flow cytometer. Annexin V-Alexa647 and antibodies against P-selectin (CD62P-Alexa647), glycoprotein Ib (CD42b-PE), glycoprotein IIb/IIIa (CD61-PE) and its activation marker (PAC1-FITC) were from Sony Biotechnology (San Jose, CA, USA). Cysteine-containing version of collagen-related peptide (CRP) was custom-synthesized and purified by VCPBIO (Shenzhen, China) and then crosslinked in-house. All other reagents were from Sigma-Aldrich (St Louis, MO, USA). Along with the assessment of expression of the main glycoproteins, we also evaluated the size and granularity parameters of platelets by estimation of forward light scattering (FSC) and side light scattering (SSC) of the cells.

#### Thromboelastography

Thromboelastography was performed with a TEG 5000 thromboelastograph (Haemoscope Corp, Niles, IL, USA) using 340 μl whole citrated blood pre-activated with kaolin and recalcified with 20 μl of 0.2 M CaCl2. The following parameters were included into the analysis: R—the time until the fibrin formation start; K—the time needed to reach a certain level of clot strength; alpha (α) angle—the rapidity of fibrin build-up and cross-linking (clot strengthening); MA—maximum amplitude, a direct function of the maximum dynamic properties of the fibrin-platelet aggregate.

#### Thrombodynamics

Thrombodynamics assay was performed with a thrombodynamics analyzer and kit (HemaCore LLC, Moscow, Russia) in platelet-free plasma (PFP), obtained by serial centrifugation at 1600g for 15 min and at 10,000g for 5 min. This method is based on registering spatial fibrin clot growth after activation of clotting in a thin layer of plasma after contact with an immobilized tissue factor-bearing surface [16, 18, 21, 24, 28–32]. The process of clot growth was registered by serial photography during the test. Based on the photos, a plot of clot growth versus time was obtained. In some cases, spontaneous clotting (clot formation in the cuvette space not associated with the main clot growth) occurred and was described with a spontaneous clotting plot. The following parameters were used for analysis: Tlag—lag time, the time to reach half-maximal light scattering near the activator; Vi—initial clot growth rate, the average rate of clot growth calculated on the interval 2–6 min after the beginning of clot growth; V—clot growth rate, the average rate of clot growth calculated in the interval 15–25 min after the beginning of clot growth [Tlag + 15 min; Tlag + 25 min]; if V could not be calculated on this interval because of the presence of spontaneous clots, it was calculated on the interval [Tsp−10min, Tsp]; CS_30—the clot size at the 30th minute of measurement; Tsp—the time needed for spontaneous clotting to fill 5% of the analyzed cuvette area. Parameter D was calculated as the maximal light scattering of the clot.

### Single nuclear polymorphisms (SNP) genotyping

We studied SNPs of 7 genes of the blood coagulation system associated with an increased risk of thromboembolism and with resistance to antiplatelet agents, mainly to aspirin: G20210A mutation of the prothrombin FII gene (rs1799963), FV Leiden mutation (rs6025), FXIII V34L polymorphism of F13A1 gene (rs5985), platelet GP Ia/IIa ITGA2 gene polymorphism (rs1126643), platelet GP IIb/IIIa ITGB3 gene polymorphism (rs5918), platelet GP Ib GP1BA gene polymorphism (rs6065), platelet GPVI GP6 gene polymorphism (rs1613662) and plasminogen activator inhibitor-1 (PAI-1) SERPINE-1 gene polymorphism (rs1799889).

For patient genotyping, DNA was isolated from EDTA-stabilized peripheral venous blood using the QIAmp DNA Blood Mini Kit (QIAGEN) and the QIAcube automated station (QIAGEN) according to the manufacturer’s recommendations.

Genotyping was performed using real-time PCR using SNP reagent kits (DNA technology, Moscow, Russia) according to the manufacturer’s recommendations.

### Statistical analysis

Mean (±SD) and Median (min-max) were used to estimate the results in case of normal or not normal distributions, respectively. Mann-Whitney U-test (non-related samples), Wilcoxon sighed rank test (related samples) for continuous data or Fisher’s exact test for categorical data were used for statistical analysis. To estimate the strength of the correlations Spearman correlation coefficient was calculated. Results were considered significant if p<0.05. Statistical analysis was performed using Origin Pro 2018 (OriginLab Corp., Northampton, MA, USA) software.

### Potential sources of bias

The main potential sources of bias were the heterogeneity of patients’ anamnesis. To address these potential sources of bias we compared the groups with outcomes according to their clinical characteristics prior to the main analysis.

## Results

### Study population characteristics

In the study 37 patients with IE and 53 healthy volunteers were included. The number of cases in the area during the study period determined the sample size. We had no loss of follow-up during the study.

Basic characteristics for both groups are shown in Table 1.

**Table 1:** Clinical characteristics of cohorts.

	Patients with IE (n = 37)	Control group (n = 53)	p*
Age, Me(IQR)	62 (42–73)	33 (25–43)	<0.001
Sex (male/female), N (%)	23 (62)/14(38)	17 (32)/36 (68)	<0.001
Body mass index (BMI), M±SD	26.3±6.9	23.3±4.1	NS
Haemoglobin, M±SD	100,7±24,5	134,0±14,0	<0.001
WBC, 10^9^/L, M±SD	10,73±4,63	5.8±1.4	<0.001
PLT, 10^9^/L, M±SD	221,81±102,66	243.7±46.0	NS

* For comparison of continuous data Mann-Whitney U-test was used. For comparison of categorical data Fisher’s exact test was used.

Me—Median, IQR—interquartile range, M—Mean, SD—standard deviation, IE -infective endocarditis, BMI—body mass index, WBC—white blood cells concentration, PLT—platelet concentration, n—sample size.

Age and gender distributions were significantly different between groups. However, the laboratory methods used in the study are non-sensitive to age or gender [33]. The parameters of the CBC differed significantly between patients with IE and healthy controls, namely: concentration of leukocytes was significantly increased, concentrations of hemoglobin and platelets were significantly decreased.

The median duration of the observation per patient was 25 days (IQR 15–45). Thirteen patients (35%) had EE either on admission or under antibiotic (AB) therapy. Ten patients already had emboli on admission (6 had multifocal septic pulmonary embolism, 1 had spondylodiscitis, 1 had spleen embolism, 1 had myocardial infarction and 1 had spleen and kidney arteries embolism), in 5 patients embolization has worsened throughout the hospital stay (3 had an increase in the percentage of embolization of an already affected organ and 2 patients had cerebral embolism in addition to the existing localization). In three patients who had no emboli on admission embolization was detected during AB therapy (1 had cerebral embolism, 1 had myocardial infarction and 1 had spondylodiscitis). In 9 patients (32%) IE was lethal during their stay in the hospital. Three more patients died during 1 year after hospital discharge. In-hospital lethal outcomes were not associated with embolization (only 3 patients out of 9 (33%) had emboli in the cohort with fatal IE, while in discharged patients 10 out of 28 (35%) had emboli). Clinical characteristics for patients with IE, divided into subgroups according to EE presence and fatal in-hospital IE, are summarized in Table 2.

**Table 2:** Clinical characteristics of groups according to outcomes.

	All Patients (n = 37)	IE with EE (n = 13, 35.1%)	IE without EE (n = 24, 64.9%)	p*	Fatal IE (n = 9, 24.3%)	Non-fatal IE (n = 28, 75.6%)	p*
Sex (male/ female), n (%)	23/14(62.16)	9/4(69.23)	14/10(58.33)	NS	5/4(55.5)	18/10(64.00)	NS
Age, years Me(IQR)	62 (42–73)	49(35–68)	66(54–80)	0.016	72(49–75)	60(37–70)	NS
History of cardiovascular diseases, n (%)	27(72.97)	9(69.23)	18(75.00)	NS	5(55.55)	22(78.57)	NS
Ischaemic heart disease (CAD), n (%)	13(35.14)	4(30.77)	9(37.50)	NS	3(33.33)	10(35.71)	NS
Atrial fibrillation, n (%)	11(29.73)	3(23.08)	8(33.33)	NS	5(55.55)	6(66.66)	NS
Arterial hypertension, n (%)	25(67.57)	7(53.85)	18(75.00)	NS	7(77.77)	18(64.28)	NS
Heart failure, n (%)	22(59.46)	6(46.15)	16(66.67)	NS	5(55.55)	17(60.71)	NS
Duration of IE (days), Me(IQR)	39(17–77)	50(16–80)	38(19–70)	NS	16(8–47)	48(28–81)	0.033
Duration of hospitalization (days), Me(IQR)	25(15–45)	25(9–44)	26(17–46)	NS	17(9–25)	27(16–48)	NS
Length of stay in the ICU (days), Me(IQR)	0(0–4)	4(0–5)	0(0–2)	NS	4(1–13)	0(0–3)	0.004
Diabetes mellitus, n (%)	8(21.62)	2(15.38)	6(25.00)	NS	2(22.22)	6(21.43)	NS
Chronic autoimmune disease, n (%)	3(8.11)	1(7.69)	2(8.33)	NS	0(0.00)	3(10.71)	NS
Cancer, n (%)	4(10.81)	1(7.69)	3(12.50)	NS	1(11.11)	3(10.71)	NS
HIV, n (%)	2(5.41)	1(7.69)	1(4.17)	NS	0(0.00)	2(7.14)	NS
Hepatitis B, n (%)	1(2.7)	0(0.00)	1(4.17)	NS	1(11.11)	0(0.00)	NS
Hepatitis C, n (%)	8(21.62)	5(38.46)	3(12.50)	NS	1(11.11)	7(25.00)	NS
Severe liver disease (cirrhosis), n (%)	4(10.81)	0(0.00)	4(16.67)	NS	1(11.11)	3(10.71)	NS
COPD/ Asthma, n (%)	2(5.41)	1(7.69)	1(4.17)	NS	1(11.11)	1(3.57)	NS
Previous Pulmonary embolism, n (%)	1(2.70)	0(0.00)	1(4.17)	NS	0(0.00)	1(3.57)	NS
Previous Stroke /TIA, n (%)	6(16.22)	2(15.38)	4(16.66)	NS	2(22.22)	4(14.29)	NS
Alcohol abuse, n (%)	9(24.32)	4(30.77)	5(20.83)	NS	2(22.22)	7(25.00)	NS
CKD, n (%)	13(35.14)	5(38.46)	8(33.33)	NS	6(66.66)	7(25.00)	NS
Index Charlson^b^, M±SD	6.91±3.71	5.61±3.59	7.63±3.65	NS	8.00±3.43	6.57±3.79	NS
body mass index (BMI), M±SD	26.30±6.86	27.95±10.46	25.40±3.76	NS	29.04±9.69	25.42±5.63	NS
Predisposing factors							
Previous endocarditis, n (%)	6(16.22)	3(23.08)	3(12.50)	NS	0(0)	6(21.42)	NS
Hypertrophic cardiomyopathy, n (%)	1(2.70)	0(0.00)	1(4.17)	NS	0(0.00)	1(3.57)	NS
Rheumatic heart disease, n (%)	3(8.11)	0(0.00)	3(12.5)	NS	0(0.00)	3(10.71)	NS
Degenerative valve disease, n (%)	10(27.03)	2(15.38)	8(33.33)	NS	3(33.33)	7(25.00)	NS
Myxomatous degeneration/mitral valve prolapse, n (%)	4(10.81)	1(7.69)	3(12.50)	NS	0(0.00)	4(14.30)	NS
Valve calcification, n (%)	1(2.7)	1(7.69)	0(0.00)	NS	0(0.00)	1(3.57)	NS
Congenital heart defect, n (%)	4(10.81)^a^	1(7.69)	3(12.5)	NS	0(0.00)	4(14.28)	NS
Prosthetic valves, n (%)	9(24.32)	2(15.38)	7(29.17)	NS	2(22.22)	7(25.00)	NS
Mechanical prosthesis, n (%)	6(16.22)	2(15.38)	4(16.66)	NS	1(11.11)	5(17.86)	NS
Bioprosthesis, n (%)	3(8.11)	0(0.00)	3(12.50)	NS	1(11.11)	2(7.14)	NS
Pacemaker, n (%)	1(2.70)	1(7.69)	0(0.00)	NS	1(11.11)	0(0.00)	NS
Intravenous drug dependency, n (%)	7(18.92)	5(38.46)	2(8.33)	0.025	0(0.00)	7(25.00)	NS
Laboratory parameters							
Haemoglobin, M±SD	100.68±24.47	95.69±33.36	103.37±18.29	NS	101.77±29.57	100.32±23.21	NS
WBC, M±SD	10.73±4.63	12.60±5.35	9.71±3.93	0.029	10.71±4.81	10.73±4.66	NS
Platelets, M±SD	221.81±102.66	232.46±98.43	216.04±106.50	NS	180.44±75.74	235.10±107.70	NS
Platelets < 100000, n (%)	6(16.22)	1(7.69)	5(20.83)	NS	2(22.22)	4(14.28)	NS
Characteristics of vegetations							
Left-side IE, n (%)	26(70.27)	7(53.84)	19(79.16)	NS	8(88.88)	17(60.71)	NS
Right-side IE, n (%)	11(29.73)	6(46.15)	5(20.83)	NS	1(3.57)	10(35.71)	NS
Vegetations > 10 mm, n (%)	20(55.56)	6(50.00)	14(58.33)	NS	3(33.33)	17(60.71)	NS
Vegetations > 13 mm, n (%)	18(48.65)	7(53.85)	11(45.83)	NS	2(22.22)	15(53.57)	NS

IE—Infective endocarditis, Me—median, IQR—interquartile range, M—mean, SD—standard deviation, EE—embolic events, CAD—coronary artery disease, HIV—human immunodeficiency virus, COPD—Chronic obstructive pulmonary disease, TIA—transient ischemic attack, CKD—chronic kidney disease, WBC—white blood cells, NS—non-significant difference.

*For comparison of continuous data Mann-Whitney U-test was used. For comparison of categorical data Fisher’s exact test was used. In the case of zero values in any of the groups, the reliability of difference was additionally verified by the χ^2^ Pearson test.

^a^Including 2 bicuspid aortic valves, 1 tetralogy of Fallot, 1 patent foramen ovale.

^b^Charlson comorbidity scale [34].

The difference in clinical parameters between the groups according to outcomes was higher number of intravenous drug addicts in the group with embolic events. Patients who subsequently died stayed in ICU for a longer time. We observed no other clinically relevant and significant difference in clinical parameters neither in groups with/without EE, nor in groups with/without fatal outcome.

Results of bacteriological blood studies are presented in S1 Table. No significant difference was found either between EE and no-EE groups, or between fatal/non-fatal IE groups according to pathogen type. Five patients with EE and 12 patients without EE had sterile blood samples. In group of fatal IE 8 patients had sterile blood samples while in non-fatal group 9 patients had sterile blood samples. In these cases the definite IE diagnosis was made based on clinical Duke criteria: 1 major and 3 minor criteria.

### Anticoagulant and antiplatelet therapy

Some of the patients received anticoagulant, antiplatelet or combined therapy upon admission to the hospital due to the presence of concomitant diseases (S2 Table). While in the hospital, therapy agents could be replaced due to the upcoming surgical treatment of IE (mainly replacement of warfarin/direct oral anticoagulants (DOAKs) with LMWH), or prescribed due to the newly diagnosed concurrent conditions. The groups with / without EE and fatal / non-fatal IE did not differ in the prescribed therapy.

### Laboratory assay data

We compared all laboratory hemostasis parameters of IE patients with healthy volunteers at admission.

**Fig 1:**
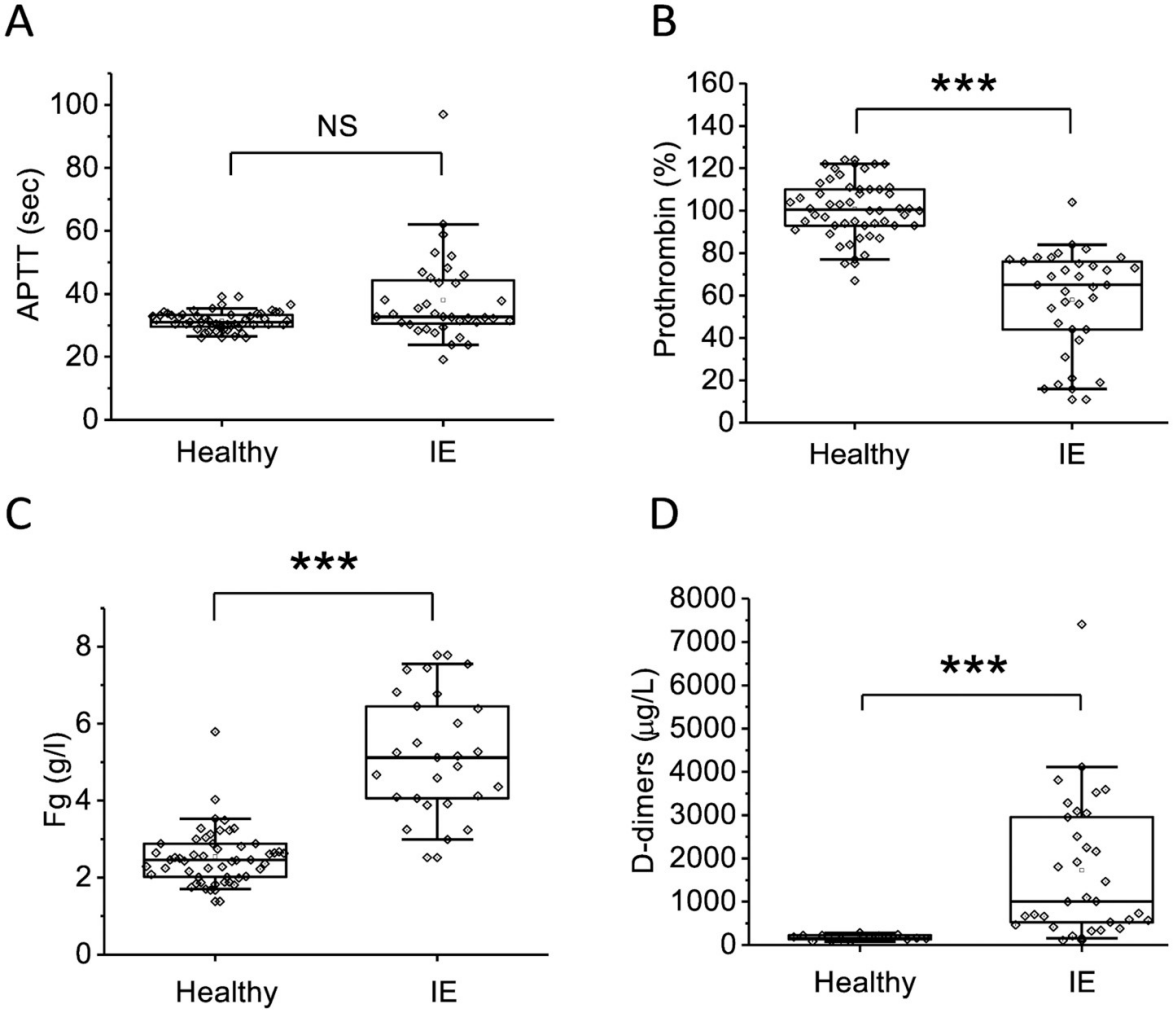
Parameters of routine coagulation tests in IE patients and healthy volunteers at admission. A—APTT (sec), B—prothrombin (%), C—fibrinogen (g/l), D—D-dimers (μg/l). The box plots indicate the following parameters: the mean value (the dot inside the box), the median (the horizontal line inside the box), the 25^th^ and 75^th^ percentiles (the bottom and top of the box, respectively) and 5^th^ and 95^th^ percentiles (the ends of the whiskers). Groups were compared according to Mann-Whitney test; *p<0.05, **p<0.01, ***p<0.001, NS—not significant.

We observed changes in the parameters of routine hemostasis tests in patients with infective endocarditis compared with healthy volunteers. The main distributions of APTT did not differ between groups, however, a pronounced elongation of APTT was observed in some patients (Fig 1A). All patients showed a significant decrease in prothrombin (Fig 1B), increase in fibrinogen (Fig 1C) and elevation of D-dimer level (Fig 1D). The data of standard tests indirectly indicate the presence of hypercoagulation against the background of inflammation, which is evidenced by elevation of D-dimers and fibrinogen.

**Fig 2:**
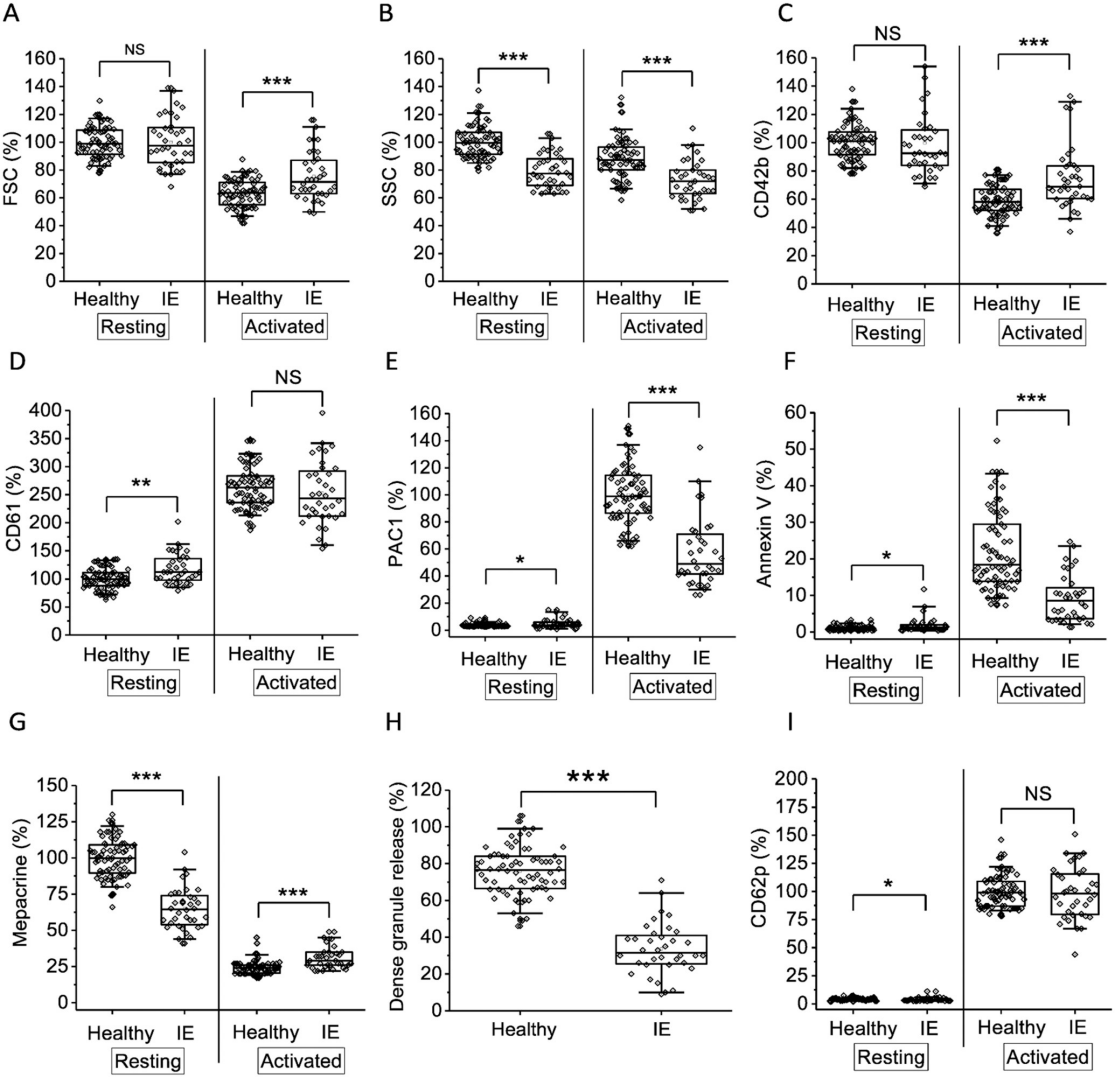
Parameters of the platelet functional activity in IE patients and healthy volunteers at admission. Platelets of 35 IE patients and 53 adult healthy volunteers were characterized by flow cytometry before and after stimulation, as described under “Materials and methods”. A—FSC; B—SSC; C—GPI (CD42b); D—total GPIIb/IIIa (CD61); E—activated GPIIb/IIIa (PAC1); F—amount of PS+ platelets (annexin V), G—dense granule amount (mepacrine uptake); H—dense granule release upon activation (calculated as the difference between resting and activated platelets mepacrine uptakes); I—P-selectin of alpha-granules. The box plots indicate the following parameters: the mean value (the dot inside the box), the median (the horizontal line inside the box), the 25^th^ and 75^th^ percentiles (the bottom and top of the box, respectively) and 5^th^ and 95^th^ percentiles (the ends of the whiskers). Groups were compared according to Mann-Whitney test; *p<0.05, **p<0.01, ***p<0.001, NS—not significant.

The size of patients’ platelets estimated by FSC parameter did not differ from the healthy volunteers before the activation. However, after platelets were activated the size was increased compared to healthy group (Fig 2A). Granularity estimated by SSC parameter was decreased both in resting and in activated platelets of patients (Fig 2B). The level of GPIb expression was elevated in platelets of patients after activation (Fig 2C), while the level of total GPIIb/IIIa on the opposite was increased in resting platelets of patients (Fig 2D). The expression of activated GPIIb/IIIa estimated with PAC-1 was decreased in platelets of patients compared to platelets of healthy volunteers (Fig 2E), as was the amount of phosphatidylserine positive (PS+) platelets upon activation (Fig 2F). The amount of mepacrine uptake by resting platelets is lower in patients than in healthy volunteers (Fig 2G) which subsequently leads to reduced dense granule release (Fig 2H), calculated as the difference between resting and activated platelets mepacrine uptakes. The amount of P-selectin representing platelet α-granules did not differ between patients and healthy volunteers (Fig 2I). Thus, according to the results of flow cytometry, it can be concluded that in patients with infective endocarditis, platelets are pre-activated in the vascular bed, which then results in their refractoriness when activated by natural agonists.

**Fig 3:**
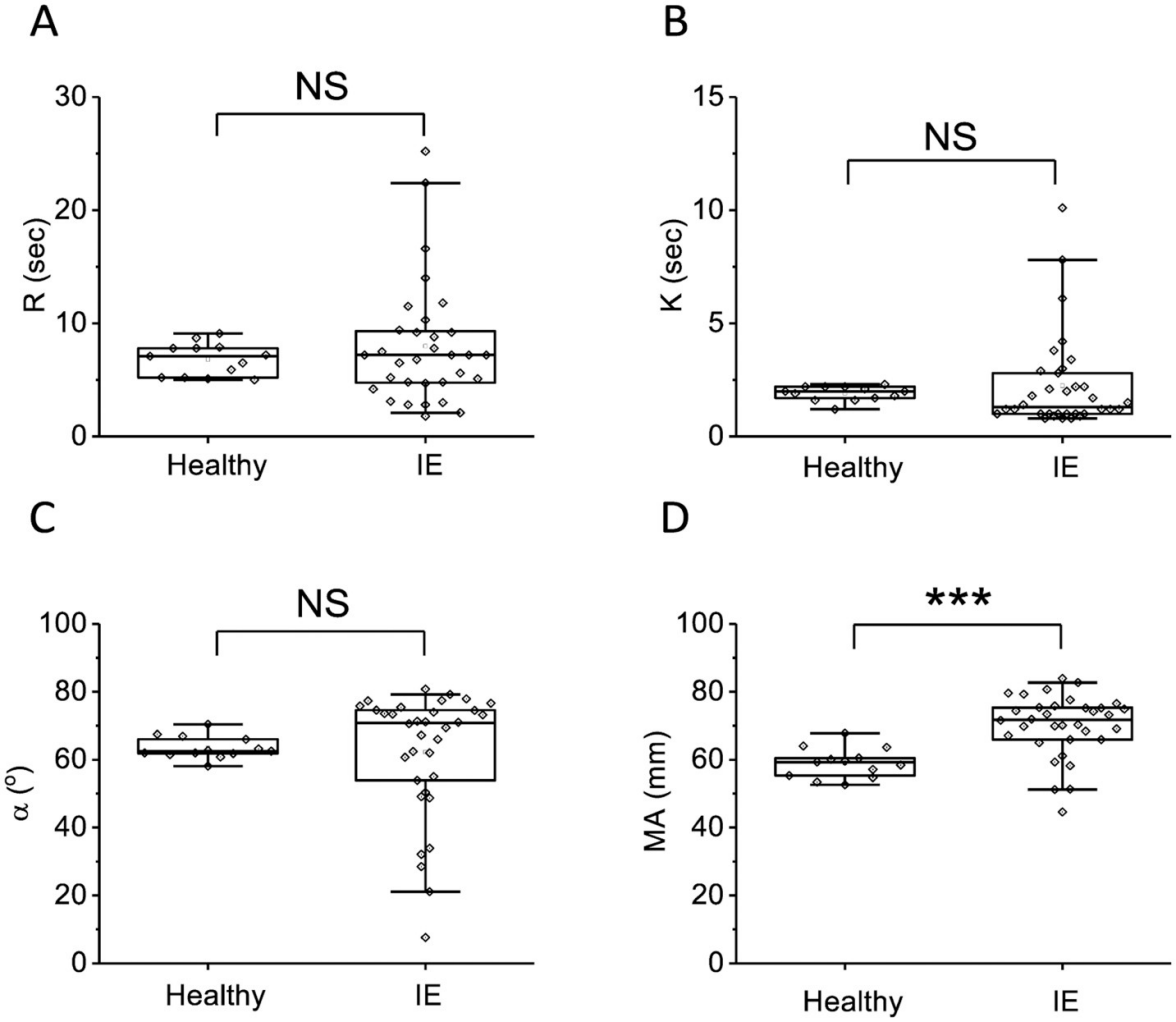
Parameters of the TEG in IE patients and healthy volunteers at admission. A—R (sec), B—K (sec), C—α (°), D—MA (mm). The box plots indicate the following parameters: the mean value (the dot inside the box), the median (the horizontal line inside the box), the 25^th^ and 75^th^ percentiles (the bottom and top of the box, respectively) and 5^th^ and 95^th^ percentiles (the ends of the whiskers). Groups were compared according to Mann-Whitney test; *p<0.05, **p<0.01, ***p<0.001, NS—not significant.

Parameters of TEG in patients did not significantly differ from the healthy volunteers, except for the fibrinogen-dependent parameter MA (Fig 3D).

**Fig 4:**
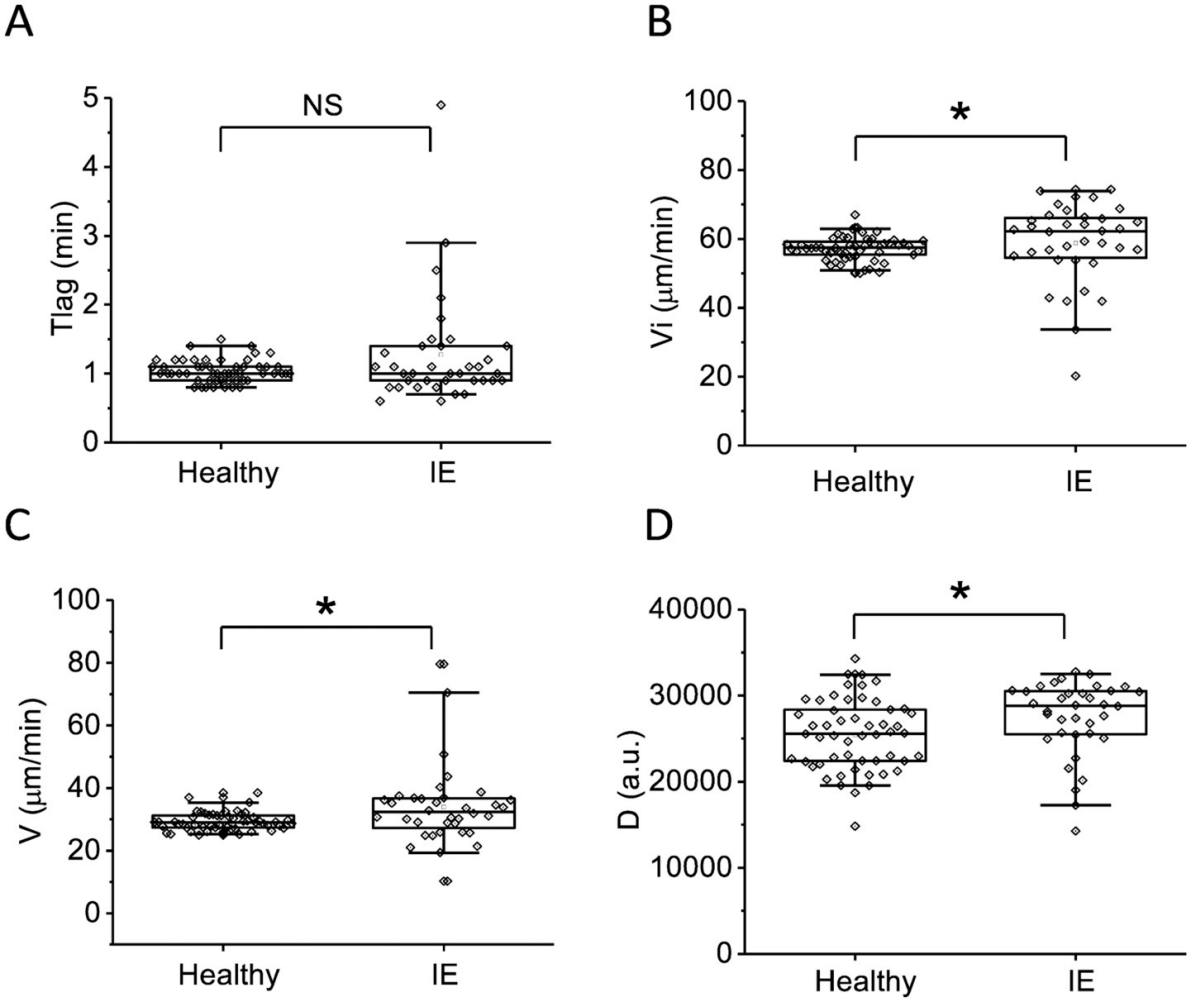
Parameters of the thrombodynamics in IE patients and healthy volunteers at admission. A—Tlag (min), B—Vi (μm/min), C—V (μm/min), D—D (a.u.). The box plots indicate the following parameters: the mean value (the dot inside the box), the median (the horizontal line inside the box), the 25^th^ and 75^th^ percentiles (the bottom and top of the box, respectively) and 5^th^ and 95^th^ percentiles (the ends of the whiskers). Groups were compared according to Mann-Whitney test; *p<0.05, **p<0.01, ***p<0.001, NS—not significant.

Parameters of thrombodynamics were shifted towards hypercoagulation. No significant difference between patients and healthy volunteers was observed for Tlag (Fig 4A), both initial and stationary clot growth rates (Fig 4B and 4C) were elevated. Eight patients (23%) had spontaneous clotting, while no healthy volunteers had spontaneous clotting. Parameter D was elevated in IE patients compared to healthy volunteers (Fig 4D).

**Fig 5:**
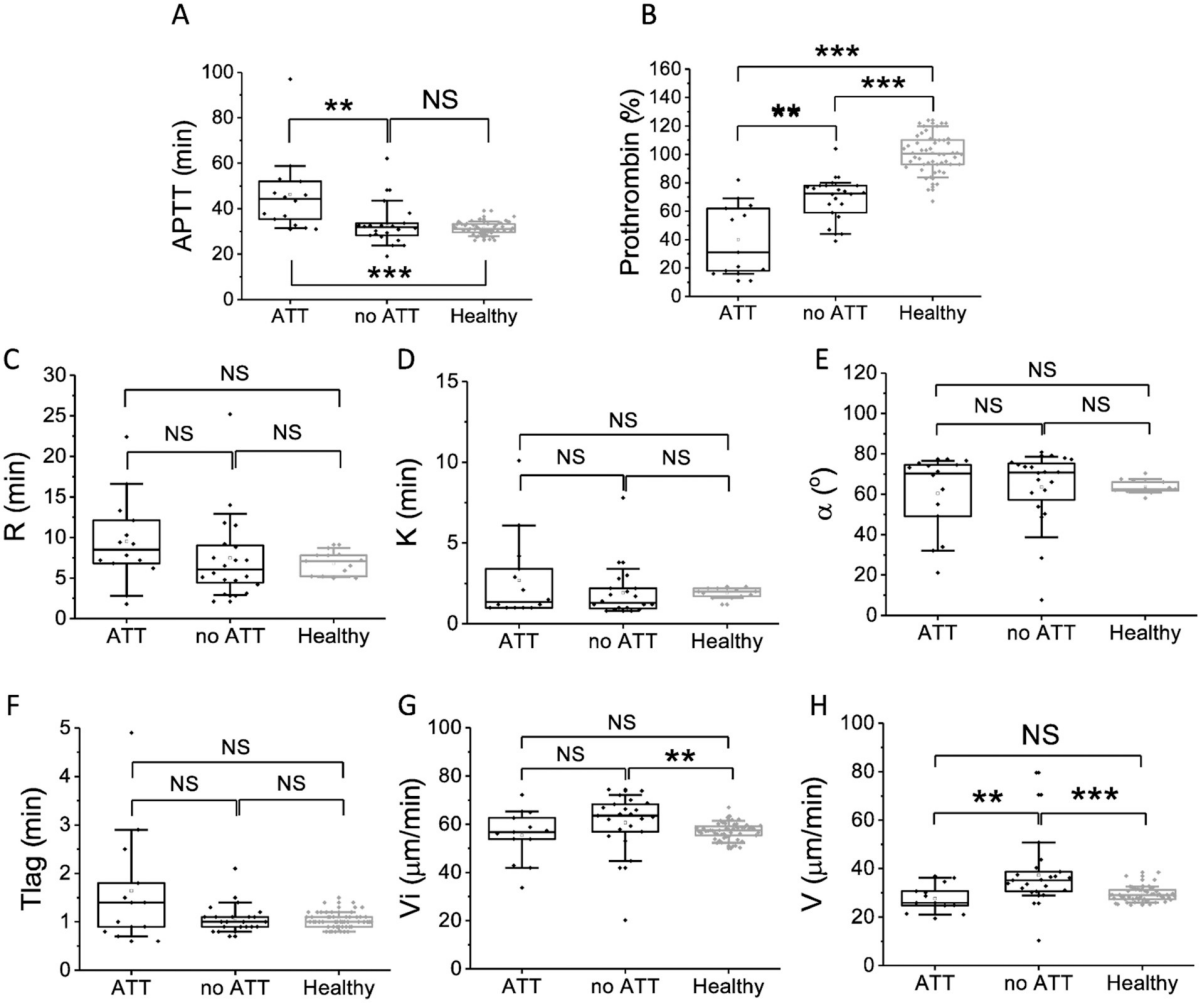
Coagulation parameters in patients receiving and not receiving antithrombotic (ATT) therapy at admission (with anticoagulants or antiplatelet agents). A—APTT (min), B—Prothrombin (%), C—R in TEG (min); D—K in TEG (min), E—α in TEG (min), F—Tlag in thrombodynamics (min), G—Vi in thrombodynamics (μm/min), H—V in thrombodynamics (μm/min). The box plots indicate the following parameters: the mean value (the dot inside the box), the median (the horizontal line inside the box), the 25^th^ and 75^th^ percentiles (the bottom and top of the box, respectively) and 5^th^ and 95^th^ percentiles (the ends of the whiskers). Groups were compared according to Mann-Whitney test; *p<0.05, **p<0.01, ***p<0.001, NS—not significant.

Since we observed a significant elongation of APTT and a decrease in prothrombin (Fig 1), as well as hypocoagulant box tails of the R, K and alpha parameters in TEG (Fig 3), elongation of Tlag and a decrease in the clot growth rates Vi and V in thrombodynamics (Fig 4), we decided to check whether the observed tendencies are a consequence antithrombotic therapy (ATT) or indicators of the consumption coagulopathy initial stages. We compared these parameters in the groups of patients receiving ATT with anticoagulants or antiplatelet agents or in the groups receiving only anticoagulants (AK) with the parameters of patients who did not receive therapy and with healthy donors (Fig 5 and S1 Fig). It was found that the elongation of APTT occurred mostly due to the previously prescribed ATT (Fig 5A). However, there still were some patients who had APTT elongation despite absence of therapy. Prothrombin was decreased both in ATT-takers and non-takers, indicating the possibility consumption coagulopathy in most patients [35] (Fig 5B). Hypocoagulant values in TEG and Vi or V in thrombodynamics did not depend on the therapy prescription (Fig 5C–5E, 5G and 5H). Elongation of Tlag was associated with ATT, but the difference was significant only for AK therapy (S1F Fig). In the group of patients who did not receive ATT, hypercoagulation in thrombodynamics was more pronounced (Fig 5G and 5H).

As for inter-assay correlations, we observed significant strong correlation between Tlag in thrombodynamics and prothrombin (RSpearman = 0.78, p<0.001) and some weak to moderate correlations between fibrinogen-dependent parameters D in thrombodynamics, MA in TEG and fibrinogen concentration (S2A, S2B and S2D Fig). MA in TEG also correlated with PAC1 amount on non-activated platelets (S2C Fig).

### Dynamics of laboratory data

No significant dynamics was observed in parameters of routine coagulation tests during hospitalization (S3 Fig). Platelet functional activity parameters showed no significant dynamics too (S4 Fig), except for dense granule release, which was increased on 14 day of in-hospital stay (S4G Fig). We observed a significant elongation of R in thromboelastography (S5A Fig) and decrease in clot growth rate Vi in thrombodynamics (S6B and S6C Fig) which characterizes the tendency towards normalization of coagulation in patients with IE as the disease is treated.

### Association of laboratory data with outcomes

The only parameter that was associated with embolization was elongation of R in thromboelastography (S3 Table). However, the difference between R distributions in groups was only slight, though significant. No association with embolization was found in parameters of standard coagulation tests, platelet functional activity or thrombodynamics. Patients with lower fibrinogen and fibrinogen-related parameter D in thrombodynamics were more likely to die, which indicates the possible presence of consumption coagulopathy in such patients at the time of hospital admission. Insufficient sample size did not allow to reliably assess the association between the coagulation tests results immediately before death with a fatal outcome.

### SNP genotyping

We performed SNP genotyping in 29 patients. No statistically significant differences were identified between the EE/no EE and fatal/non-fatal IE groups for rs5918, rs1126643, rs1613662, rs1799963, rs6025, rs1799889, rs5985 (S4 Table).

## Discussion

In our study, we assessed the laboratory parameters of hemostasis in patients with IE using routine and global tests of hemostasis and genetic polymorphisms.

At admission, coagulation parameters in all patients were shifted towards hypercoagulation as a consequence of inflammation which was expressed in rise of fibrinogen (Fig 1), increase of MA in thromboelastography (Fig 3), increase in Vi, V and D in thrombodynamics (Fig 4). In some patients we observed signs of consumption coagulopathy [24, 35, 36] expressed in decrease in prothrombin (Fig 1) and presence of decreased values of R, K and α in TEG (Fig 3) and Vi and V in thrombodynamics in the absence of antithrombotic therapy. An increase in the concentration of D-dimers in patients compared with healthy volunteers also indicates the presence of active clotting and subsequent lysis of the formed clots (Fig 1). Platelet status was characterized by a decrease in total granularity and dense granule amount in resting platelets, pre-activation of resting platelets via GP IIb/IIIa as indicated by increased amount of PAC1 on resting platelets while some of platelet responses were decreased upon activation (Fig 2). Pre-activation of platelets in the vascular bed is also an indirect confirmation of existing hypercoagulation in patients with IE. There were no significant changes in laboratory test results as the disease progressed except for the slight tendency towards normalization according to R in TEG and Vi in thrombodynamics (S3–S6 Figs). No reliable association was found between laboratory test results and clinical outcomes, except for the significant decrease of the fibrinogen and fibrinogen-dependent parameter D in thrombodynamics in the group of fatal IE outcomes and elongation of R in TEG (S3 Table) in patients with EE. We also did not find any association between genetic polymorphisms and embolic risk or mortality in our cohort (S4 Table).

There is little data on coagulation and platelets state of patients with IE. Prolongation of APTT and decreased activity of antithrombin are associated with the progression of the disease [37]. Platelet-to-lymphocyte ratio has been reported as an independent predictor of in-hospital mortality among IE patients [38]. High mean platelet volume is associated with poor prognosis and adverse outcomes, and predicts complications including embolic events [39]. An observational study by Buyukasýk et al. [10] showed an increase in hypercoagulation markers PF1+2 and TAT and platelet activation markers β-TG and platelet factor 4 (PF4) in IE patients who suffered from embolism. Laboratory parameters such as C-reactive protein, D-dimers, presence of aPL antibodies were found to be associated with EE in IE [40, 41]. However, we did not observe any significant predictive association between D-dimers and embolic events in our cohort.

The coagulation system appears to be an attractive potential target for therapy in IE. Unfortunately, because of IE rarity and heterogeneity of cohorts, quality of clinical trials in IE patients is questionable. An additional problem is that due to the high rate of cerebral embolism, up to 60% of endocarditis patients suffer intracranial hemorrhagic lesions, including microbleeds [7]. Antithrombotic therapy is thus often a difficult balancing act in which the risks of treatment can outweigh the potential benefits. Both American and European guidelines do not recommend the use of anticoagulants in the treatment of endocarditis [26, 42, 43]. However, the question remains open in situations where the patient needs to continue the already prescribed anticoagulant therapy (e.g. in case of atrial fibrillation or a mechanical heart valve presence) or in situations where the patient is unstable or in critical condition. Despite the revealed hypercoagulation in some patients in our study, it cannot be said that IE in general is characterized with severe hypercoagulation, which supports the current guidelines stating that there is no need for routine anticoagulation in patients with IE and the anticoagulants should be administered on an individual basis. Besides that no reliable association was found between laboratory test results and embolization in our cohort which also does not support the routine anticoagulant administration in patients with IE.

We were not able to identify the causative agent of infective endocarditis by culture for all patients. This fact may be related to both the limitations of the technique itself and the fact that some of the patients were admitted to the hospital already on antibiotic therapy due to fever. This fact indirectly indicates that thrombotic events in IE patients might be triggered by some bacterial constituents, such as lipopolysaccharides [44], peptidoglycan [45] and extracellular proteases [46], that may be still present in the bloodstream even after successful antibiotic therapy.

This article contains the most novel and complete information to date regarding the routine and global laboratory tests of hemostasis in patients with IE. In this study, for the first time, systemic hypercoagulation in patients with infective endocarditis is directly detected, and a tendency towards consumption coagulopathy in some patients is also shown. However, this study has some serious limitations. Firstly, it is small sample size due to the rarity of the disease. Secondly, most of the patients who developed embolism were admitted to the hospital already having emboli and vegetation in the process of destruction so that we could not observe a moment when hypercoagulation could contribute to vegetation growth or embolization. Thus, it is difficult to try to properly assess the predictive power of the laboratory tests in any way. Thirdly, it is heterogeneity of the cohort. Fourthly, it is heterogeneity of prescribed anticoagulant/antiplatelet therapy. Fifthly, asymptomatic embolism could have remained undetected, as we assessed only symptomatic embolic events.

We were unable to identify any associations between laboratory test results and clinical outcomes such as death or embolism, except for the decrease in fibrinogen in the fatal IE group. It is worth noting, however, that the issue requires further study, and perhaps an approach in which daily monitoring of hemostasis will be carried out should be implemented. This approach will allow a detailed assessment of hemostasis immediately before the onset of the outcome, rather than trying to identify associations, when testing and outcome are separated in time.

## Conclusion

Hemostasis in patients with infective endocarditis is characterized by hypercoagulation with signs of consumption coagulopathy, which is detected by routine and global hemostasis tests. Further investigation is needed to identify the association of hypercoagulation in hemostasis tests with clinical outcomes.

## Supporting information

S1 Fig Coagulation parameters in patients receiving and not receiving ancoagulant (AK) therapy at admission. A—APTT (min), B—Prothrombin (%), C—R in TEG (min); D—K in TEG (min), E—α in TEG (min), F—Tlag in thrombodynamics (min), B—Vi in thrombodynamics (μm/min), C—V in thrombodynamics (μm/min), D—D in thrombodynamics (a.u.). The box plots indicate the following parameters: the mean value (the dot inside the box), the median (the horizontal line inside the box), the 25^th^ and 75^th^ percentiles (the bottom and top of the box, respectively) and 5^th^ and 95^th^ percentiles (the ends of the whiskers). Groups were compared according to Mann-Whitney test; *p<0.05, **p<0.01, ***p<0.001, NS—not significant.
(TIF)

S2 Fig Inter-assay parameters correlations. A—Tlag in thrombodynamics vs prothrombin. B—D in thrombodynamics vs fibrinogen. C—PAC1 on non-activated platelets vs MA in TEG. D—D in thrombodynamics vs MA in TEG. To estimate the strength of the correlations Spearman correlation coefficient was calculated. Correlation was considered significant if p<0.05.
(TIF)

S3 Fig Dynamics of routine coagulation tests throughout the hospital stay. A—APTT (sec), B—prothrombin (%), C—fibtinogen (g/l), D—D-dimers (μg/l). The box plots indicate the following parameters: the mean value (the dot inside the box), the median (the horizontal line inside the box), the 25^th^ and 75^th^ percentiles (the bottom and top of the box, respectively) and 5^th^ and 95^th^ percentiles (the ends of the whiskers). Results were compared according to Wilcoxon signed rank test; *p<0.05, **p<0.01, ***p<0.001, NS—not significant.
(TIF)

S4 Fig Dynamics of platelet function parameters throughout the hospital stay. A—FSC; B—SSC; C—GPI (CD42b); D—total GPIIb/IIIa (CD61); E—activated GPIIb/IIIa (PAC1); F—amount of PS+ platelets (annexin V), G—dense granule amount (mepacrine uptake); H—dense granule release upon activation (calculated as the difference between resting and activated platelets mepacrine uptakes); I—P-selectin of alpha-granules. The box plots indicate the following parameters: the mean value (the dot inside the box), the median (the horizontal line inside the box), the 25^th^ and 75^th^ percentiles (the bottom and top of the box, respectively) and 5^th^ and 95^th^ percentiles (the ends of the whiskers). Results were compared according to Wilcoxon signed rank test; *p<0.05, **p<0.01, ***p<0.001, NS—not significant.
(TIF)

S5 Fig Dynamics of thromboelastography parameters throughout the hospital stay. A—R (sec), B—K (sec), C—α (°), D—MA (mm). The box plots indicate the following parameters: the mean value (the dot inside the box), the median (the horizontal line inside the box), the 25^th^ and 75^th^ percentiles (the bottom and top of the box, respectively) and 5^th^ and 95^th^ percentiles (the ends of the whiskers). Results were compared according to Wilcoxon signed rank test; *p<0.05, **p<0.01, ***p<0.001, NS—not significant.
(TIF)

S6 Fig Dynamics of thrombodynamics parameters throughout the hospital stay. A—Tlag (min), B—Vi (μm/min), C—V (μm/min), D—D (a.u.). The box plots indicate the following parameters: the mean value (the dot inside the box), the median (the horizontal line inside the box), the 25^th^ and 75^th^ percentiles (the bottom and top of the box, respectively) and 5^th^ and 95^th^ percentiles (the ends of the whiskers). Results were compared according to Wilcoxon signed rank test; *p<0.05, **p<0.01, ***p<0.001, NS—not significant.
(TIF)

S1 Table Bacteriological blood studies in IE patients. (DOCX)

S2 Table Anticoagulant therapy in IE patients. (DOCX)

S3 Table Association of laboratory data with outcomes. (DOCX)

S4 Table Association of SNP data with outcomes. (DOCX)
