# Supplementary figures and images for: Hypercoagulation detected by routine and global laboratory hemostasis assays in patients with infective endocarditis
Source: PLoS One. 2021 Dec 15;16(12):e0261429. doi: 10.1371/journal.pone.0261429 (PMC8673624; doi:10.1371/journal.pone.0261429)

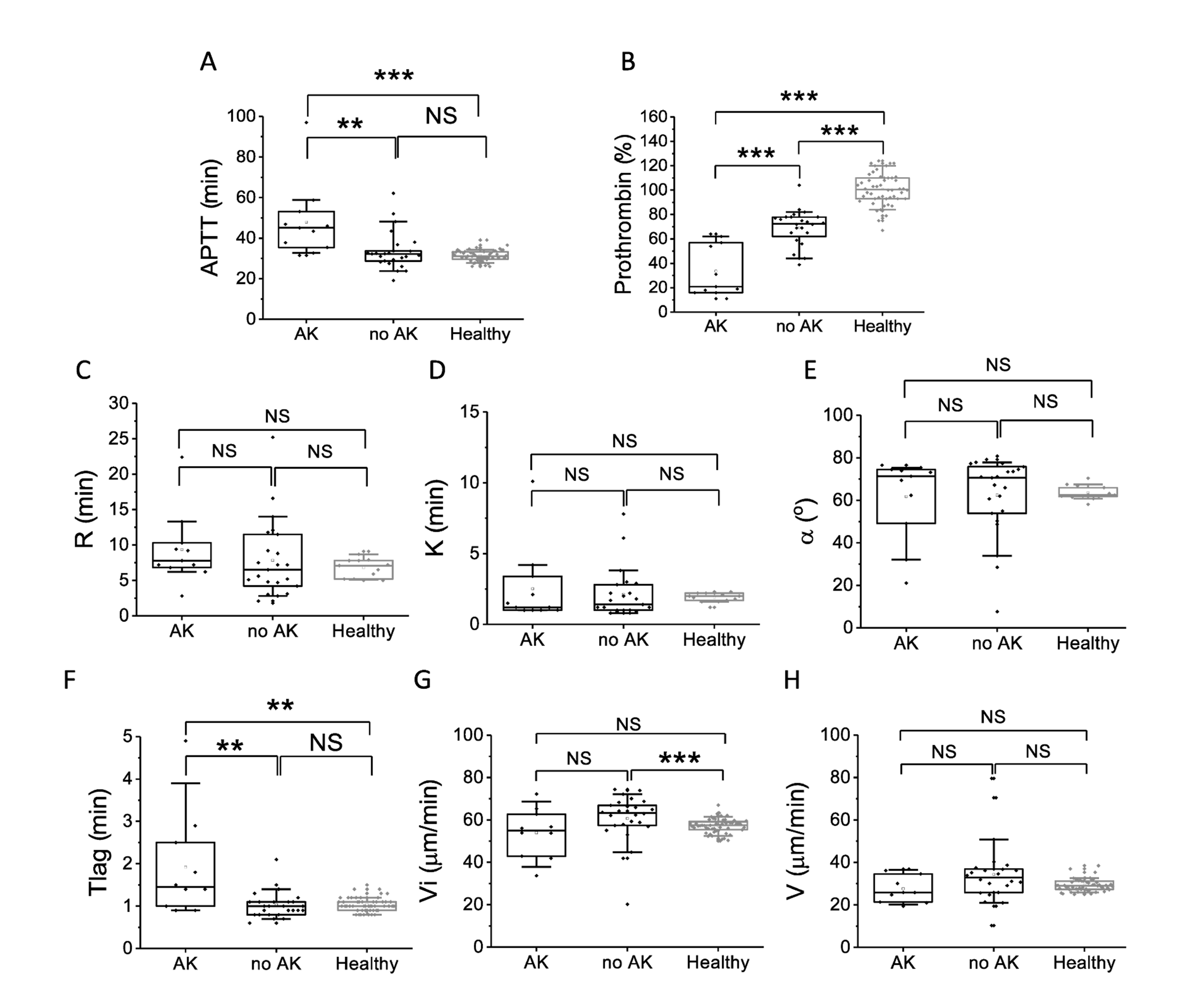

Supplement: S1 Fig — A—APTT (min), B—Prothrombin (%), C—R in TEG (min); D—K in TEG (min), E—α in TEG (min), F—Tlag in thrombodynamics (min), B—Vi in thrombodynamics (μm/min), C—V in thrombodynamics (μm/min), D—D in thrombodynamics (a.u.). The box plots indicate the following parameters: the mean value (the dot inside the box), the median (the horizontal line inside the box), the 25th and 75th percentiles (the bottom and top of the box, respectively) and 5th and 95th percentiles (the ends of the whiskers). Groups were compared according to Mann-Whitney test; *p<0.05, **p<0.01, ***p<0.001, NS—not significant. (TIF) [file pone.0261429.s001.tif]

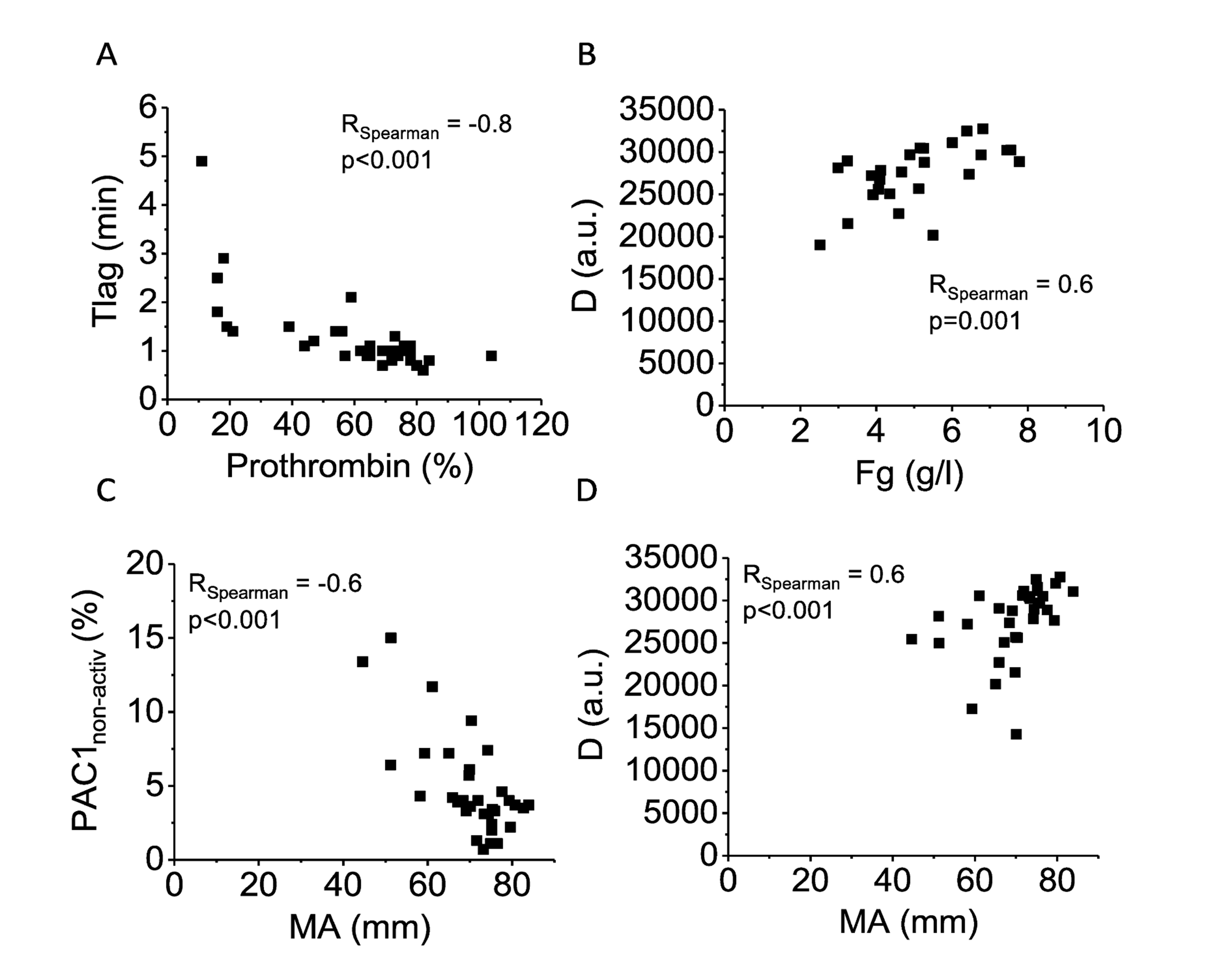

Supplement: S2 Fig — A—Tlag in thrombodynamics vs prothrombin. B—D in thrombodynamics vs fibrinogen. C—PAC1 on non-activated platelets vs MA in TEG. D—D in thrombodynamics vs MA in TEG. To estimate the strength of the correlations Spearman correlation coefficient was calculated. Correlation was considered significant if p<0.05. (TIF) [file pone.0261429.s002.tif]

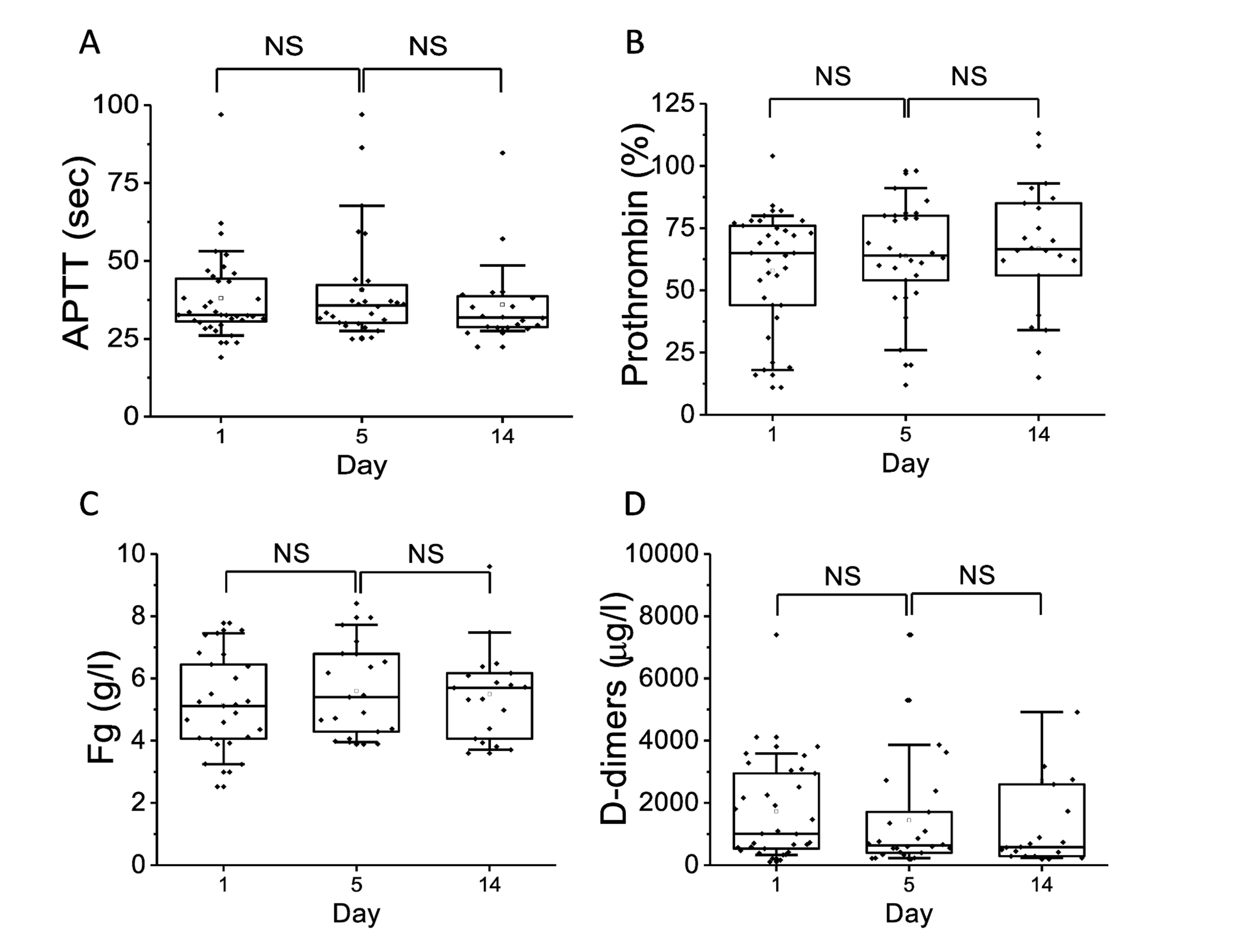

Supplement: S3 Fig — A—APTT (sec), B—prothrombin (%), C—fibtinogen (g/l), D—D-dimers (μg/l). The box plots indicate the following parameters: the mean value (the dot inside the box), the median (the horizontal line inside the box), the 25th and 75th percentiles (the bottom and top of the box, respectively) and 5th and 95th percentiles (the ends of the whiskers). Results were compared according to Wilcoxon signed rank test; *p<0.05, **p<0.01, ***p<0.001, NS—not significant. (TIF) [file pone.0261429.s003.tif]

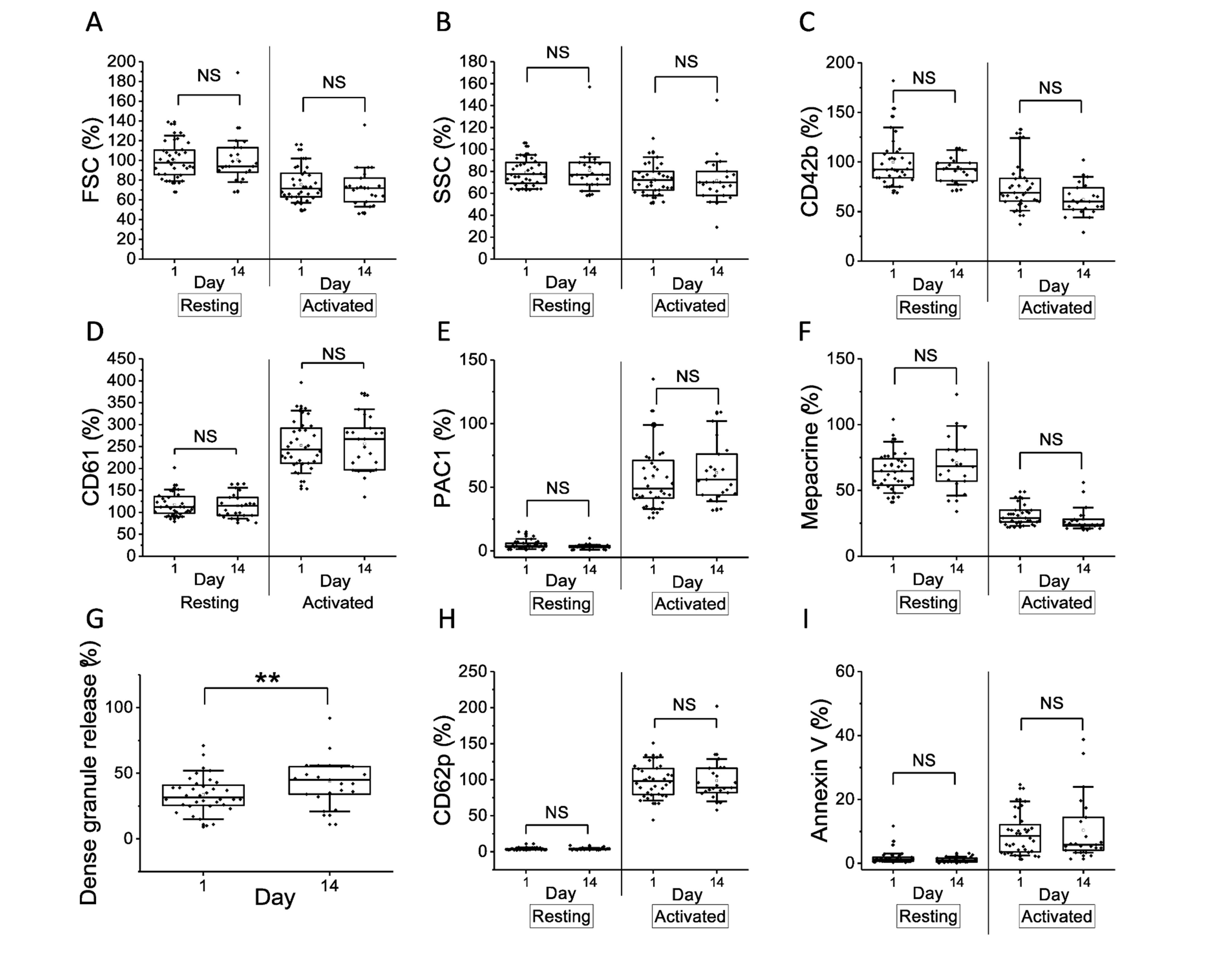

Supplement: S4 Fig — A—FSC; B—SSC; C—GPI (CD42b); D—total GPIIb/IIIa (CD61); E—activated GPIIb/IIIa (PAC1); F—amount of PS+ platelets (annexin V), G—dense granule amount (mepacrine uptake); H—dense granule release upon activation (calculated as the difference between resting and activated platelets mepacrine uptakes); I—P-selectin of alpha-granules. The box plots indicate the following parameters: the mean value (the dot inside the box), the median (the horizontal line inside the box), the 25th and 75th percentiles (the bottom and top of the box, respectively) and 5th and 95th percentiles (the ends of the whiskers). Results were compared according to Wilcoxon signed rank test; *p<0.05, **p<0.01, ***p<0.001, NS—not significant. (TIF) [file pone.0261429.s004.tif]

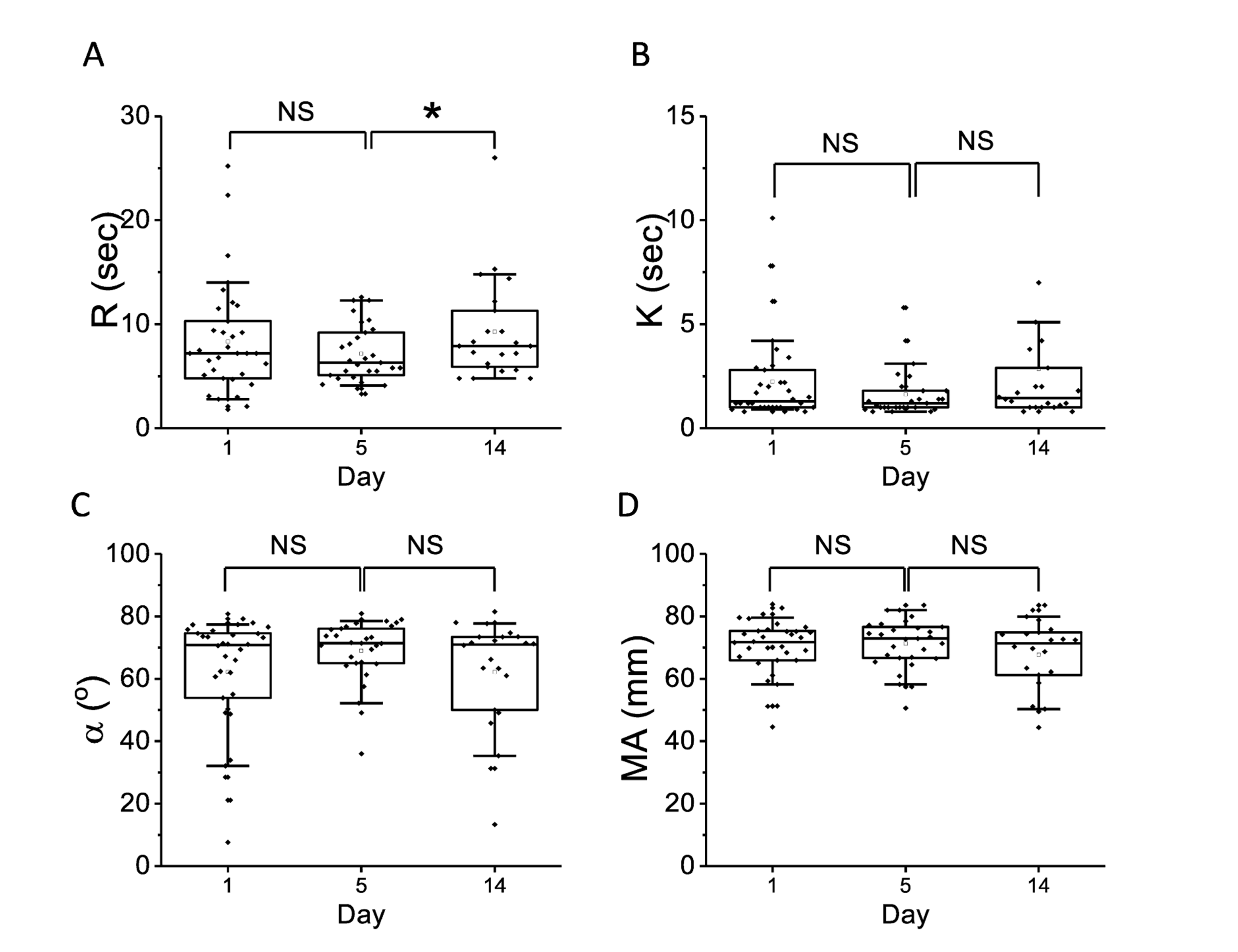

Supplement: S5 Fig — A—R (sec), B—K (sec), C—α (°), D—MA (mm). The box plots indicate the following parameters: the mean value (the dot inside the box), the median (the horizontal line inside the box), the 25th and 75th percentiles (the bottom and top of the box, respectively) and 5th and 95th percentiles (the ends of the whiskers). Results were compared according to Wilcoxon signed rank test; *p<0.05, **p<0.01, ***p<0.001, NS—not significant. (TIF) [file pone.0261429.s005.tif]

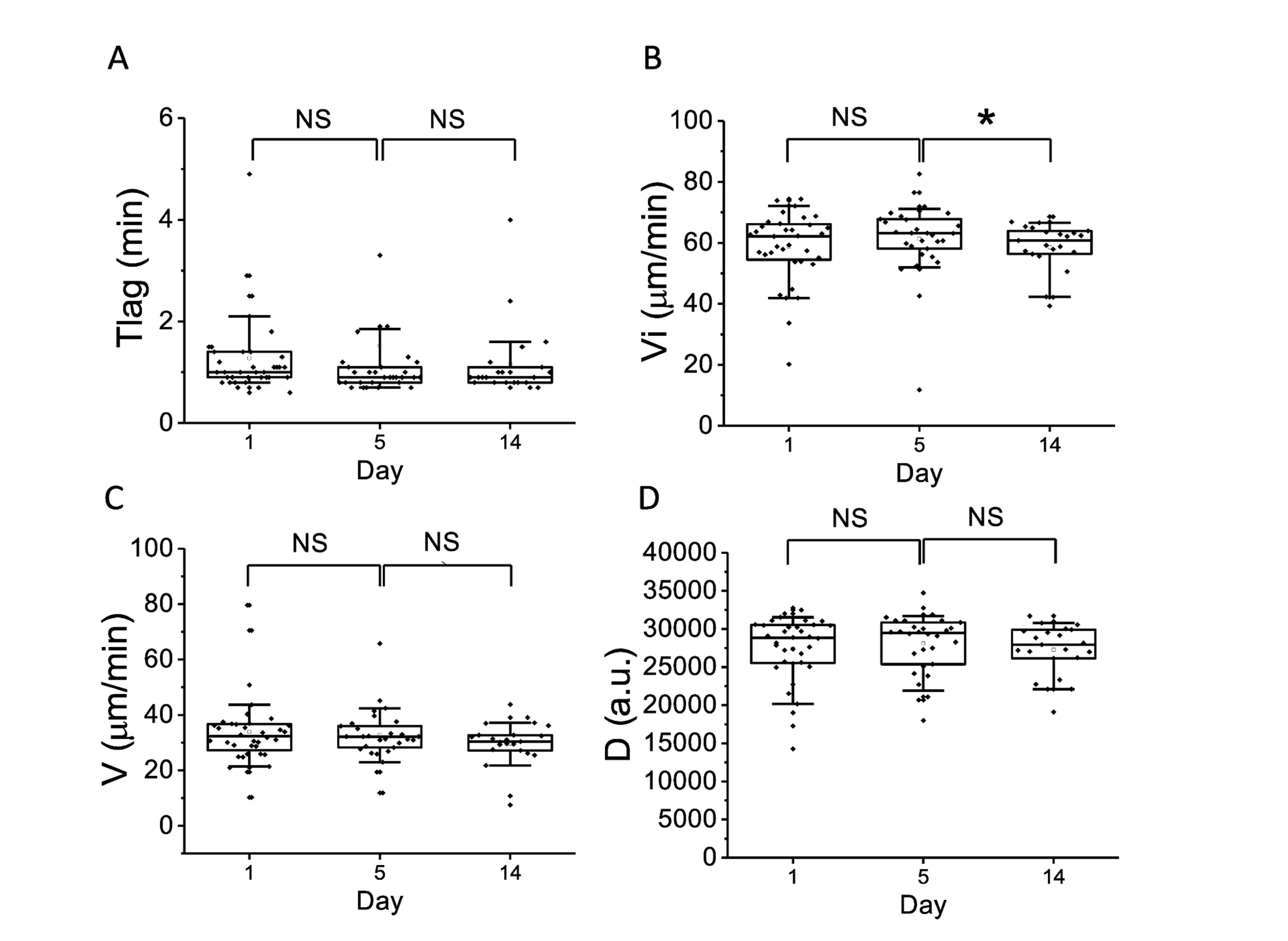

Supplement: S6 Fig — A—Tlag (min), B—Vi (μm/min), C—V (μm/min), D—D (a.u.). The box plots indicate the following parameters: the mean value (the dot inside the box), the median (the horizontal line inside the box), the 25th and 75th percentiles (the bottom and top of the box, respectively) and 5th and 95th percentiles (the ends of the whiskers). Results were compared according to Wilcoxon signed rank test; *p<0.05, **p<0.01, ***p<0.001, NS—not significant. (TIF) [file pone.0261429.s006.tif]
