# Supplementary material for: Hypercoagulation detected by routine and global laboratory hemostasis assays in patients with infective endocarditis
Source: PLoS One. 2021 Dec 15;16(12):e0261429. doi: 10.1371/journal.pone.0261429 (PMC8673624; doi:10.1371/journal.pone.0261429)
Supplement: S1 Table — (DOCX) [file pone.0261429.s007.docx]

**S1 Table. Bacteriological blood studies in IE patients.**

|  | All Patients (n=37) | IE with EE (n=13, 35.1%) | IE without EE (n=24, 64.9%) | p* | Fatal IE (n=9, 24.3%) | Non-fatal IE (n=28, 75.6%) | p* |
| --- | --- | --- | --- | --- | --- | --- | --- |
| Enterococcus faecalis or faecium **,** n (%) | 5 (13.50) | 1 (7.69) | 4 (16.67) | NS | 0 (0.00) | 5 (17.86) | NS |
| Enterobacter cloacae **,** n (%) | 1 (2.70) | 1 (7.69) | 0 (0.00) | NS | 0 (0.00) | 1 (3.57) | NS |
| Streptococcus viridans **,** n (%) | 3 (8.11) | 1 (7.69) | 2 (8.33) | NS | 0 (0.00) | 3 (10.71) | NS |
| Staphylococcus aureus**,** n (%) | 6 (16.22) | 3 (23.08) | 3 (12.50) | NS | 0 (0.00) | 6 (21.43) | NS |
| Staphylococcus haemolyticus**,** n (%) | 3 (8.11) | 2 (15.38) | 1 (4.17) | NS | 1 (11,11) | 2 (7.14) | NS |
| Staphylococcus epidermidis**,** n (%) | 1 (2.70) | 0 (0.00) | 1 (4.17) | NS | 0 (0.00) | 1 (3.57) | NS |
| Gemella haemolysans**,** n (%) | 1 (2.70) | 0 (0.00) | 1 (4.17) | NS | 0 (0.00) | 1 (3.57) | NS |
| Negative culture or absence of blood culture **,** n (%) | 17 (45.95) | 5 (38.46) | 12 (50.00) | NS | 8 (88,89) | 9 (32.14) | <0.01 |

*For comparison of categorical data Fisher’s exact test was used. In the case of zero values in any of the groups, the reliability of difference was additionally verified by the χ^2^ Pearson test. NS – non-significant difference
