# Supplementary material for: Hypercoagulation detected by routine and global laboratory hemostasis assays in patients with infective endocarditis
Source: PLoS One. 2021 Dec 15;16(12):e0261429. doi: 10.1371/journal.pone.0261429 (PMC8673624; doi:10.1371/journal.pone.0261429)
Supplement: S2 Table — (DOCX) [file pone.0261429.s008.docx]

**S2 Table. Anticoagulant therapy in IE patients.**

|  | All Patients (n=37) | IE with EE (n=13, 35.1%) | IE without EE (n=24, 64.9%) | p* | Fatal IE (n=9, 24.3%) | Non-fatal IE (n=28, 75.6%) | p* |
| --- | --- | --- | --- | --- | --- | --- | --- |
| **Antithrombotic therapy total n=24 (64,86%)** | | | | | | | |
| **Anticoagulants+Antiplatelet,** n (%) | 24 (64.86) | 8 (61.53) | 16 (66.66) | NS | 6 (66.66) | 18 (64.28) | NS |
| **Anticoagulant,** n (%) | 19 (51.35) | 7 (53.84) | 12 (50.00) | NS | 6 (66.66) | 13 (46.43) | NS |
| **Antiplatelet,** n (%) | 8 (21.62) | 2 (15.38) | 6 (25.00) | NS | 3 (33.33) | 5 (17.56) | NS |
| **Antithrombotic therapy(on admission) n=14(37,84%)** | | | | | | | |
| **VKA,** n (%) | 8(21.62) | 2(15.38) | 6(25.00) | NS | 2(22.22) | 6(21.43) | NS |
| **Dabigatran,** n (%) | 1(2.70) | 1(7.69) | 0(0.00) | NS | 0(0.00) | 1(3.57) | NS |
| **Rivaroxaban,** n (%) | 2(5.40) | 0(0.00) | 2(8.33) | NS | 1(11.11) | 1(3.57) | NS |
| **Apixaban,** n (%) | 0(0.00) | 0(0.00) | 0(0.00) | NS | 0(0.00) | 0(0.00) | NS |
| **Aspirin,** n (%) | 6(16.21) | 2(15.38) | 4(16.66) | NS | 3(33.33) | 3(10.71) | NS |
| **Clopidogrel, n (%)** | 2(5.40) | 1(7.69) | 1(4.17) | NS | 1(11.11) | 1(3.57) | NS |
| **LMWH,** n (%) | 0(0.00) | 0(0.00) | 0(0.00) | NS | 0(0.00) | 0(0.00) | NS |
| **Unfractionated,** n **(%)heparin** | 0(0.00) | 0(0.00) | 0(0.00) | NS | 0(0.00) | 0(0.00) | NS |
| **Antithrombotic therapy(during hospitalization) n=23(62,16%)** | | | | | | | |
| **VKA,** n (%) | 7(18.92) | 2(15.38) | 5(20.83) | NS | 1(11.11) | 6(21.43) | NS |
| **Dabigatran,** n (%) | 0(0.00) | 0(0.00) | 0(0.00) | NS | 0(0.00) | 0(0.00) | NS |
| **Rivaroxaban,** n (%) | 3(8.11) | 1(7.69) | 2(8.33) | NS | 0(0.00) | 3(12.00) | NS |
| **Apixaban,** n (%) | 0(0.00) | 0(0.00) | 0(0.00) | NS | 0(0.00) | 0(0.00) | NS |
| **Aspirin,** n (%) | 6(16.22) | 2(15.38) | 4(16.67) | NS | 2(16.67) | 4(16.00) | NS |
| **Clopidogrel,** n (%) | 3(8.11) | 2(15.38) | 1(4.17) | NS | 2(16.67) | 1(4.00) | NS |
| **LMWH,** n (%) | 8(21.62) | 3(23.07) | 5(20.83) | NS | 4(44.44) | 4(14.29) | NS |
| **Unfractionated heparin,** n (%) | 1(2.70) | 1(7.69) | 0(0.00) | NS | 0(0.00) | 1(4.00) | NS |

*For comparison of categorical data Fisher’s exact test was used. In the case of zero values in any of the groups, the reliability of difference was additionally verified by the χ^2^ Pearson test. NS – non-significant difference
