# Supplementary material for: Hypercoagulation detected by routine and global laboratory hemostasis assays in patients with infective endocarditis
Source: PLoS One. 2021 Dec 15;16(12):e0261429. doi: 10.1371/journal.pone.0261429 (PMC8673624; doi:10.1371/journal.pone.0261429)
Supplement: S3 Table — (DOCX) [file pone.0261429.s009.docx]

**S3 Table. Association of laboratory data with outcomes.**

| Median (IQR) | Units | IE with EE (n=13, 35.1%) | IE without EE (n=24, 64.9%) | p* | Fatal IE (n=9, 24.3%) | Non-fatal IE (n=28, 75.6%) | p* |
| --- | --- | --- | --- | --- | --- | --- | --- |
| **Routine tests** | | | | | | | |
| APTT | sec | 32.2 (31.0-35.4) | 33.7 (29.4-48.2) | NS | 31.5 (28.3-33.6) | 33.7 (31.0-46.9) | NS |
| Prothrombin | % | 65 (43-74) | 62 (44-78) | NS | 57 (39-73) | 65 (44-77) | NS |
| Fibrinogen | g/l | 5.5 (4.1-6.4) | 5.0 (3.9-6.5) | NS | 3.9 (3.3-4.6) | 5.3 (4.1-6.8) | 0.02 |
| D-dimers | µg/l | 1005 (567-2997) | 1095 (528-2509) | NS | 913 (686-2434) | 1009 (467-3042) | NS |
| **Platelet functional activity** | | | | | | | |
| FSC rest | % | 101 (79-111) | 95 (86-110) | NS | 107 (94-108) | 95 (84-115) | NS |
| FSC act | % | 73 (63-88) | 68 (63-86) | NS | 78 (66-94) | 69 (63-87) | NS |
| SSC rest | % | 82 (67-86) | 75 (70-89) | NS | 80 (75-91) | 76 (68-88) | NS |
| SSC act | % | 73 (63-78) | 71 (63-80) | NS | 72 (64-84) | 72 (63-79) | NS |
| CD42b rest | % | 99 (85-110) | 92 (82-104) | NS | 87 (78.5-101.5) | 93 (86-111) | NS |
| CD42b act | % | 74 (60-84) | 69 (61-83) | NS | 64 (56-88 | 71 (62-82) | NS |
| CD61 rest | % | 112 (100-120) | 112 (94-149) | NS | 104 (95-118) | 113 (101-145) | NS |
| CD61 act | % | 228 (212-267) | 250 (212-325) | NS | 225 (212-255) | 251 (214-303) | NS |
| PAC1 rest | % | 4.0 (3.0-7.2) | 3.7 (3.1-5.3) | NS | 3.9 (3.5-5.0) | 3.8 (3.1-6.3) | NS |
| PAC1 act | % | 46 (42-73) | 51 (41-69) | NS | 51 (39-67) | 49 (42-71) | NS |
| Mepacrine uptake rest | % | 57 (53-69) | 68 (57-78) | NS | 61 (57-70) | 67 (53-75) | NS |
| Mepacrine uptake act | % | 30 (26-36) | 27 (25-35) | NS | 27 (23-34) | 30 (26-35) | NS |
| Dense granule release | % | 28 (20-38) | 35 (28-45) | NS | 30 (25-44) | 33 (26-41) | NS |
| CD62p rest | % | 3.2 (2.7-3.7) | 3.6 (3.1-5.0) | NS | 3.7 (2.9-5.4) | 3.3 (3.0-4.1) | NS |
| CD62p act | % | 89 (78-115) | 100 (82-116) | NS | 110 (100-117) | 92 (79-111) | NS |
| Annexin V rest | % | 1.0 (0.7-1.4) | 1.3 (0.8-2.6) | NS | 0.8 (0.6-1.3) | 1.3 (0.8-2.2) | NS |
| Annexin V act | % | 6.0 (3.4-10.2) | 9.8 (4.2-14.6) | NS | 4.5 (3.3-8.2) | 9.6 (4.3-13.9) | NS |
| **Thromboelastography** | | | | | | | |
| R | sec | 9.2 (6.9-11.7) | 6.2 (3.1-8.8) | 0.045 | 6.2 (4.9-7.5) | 7.4 (4.8-11.8) | NS |
| K | sec | 2.0 (1.1-4.0) | 1.2 (1.0-2.1) | NS | 1.1 (1.0-1.3) | 1.9 (1.0-3.0) | NS |
| Α | ° | 57.3 (41.5-74.1) | 71.2 (62.4-75.4) | NS | 71.2 (69.1-73.9) | 67.7 (50.3-75.4) | NS |
| MA | mm | 74.6 (67.9-77.3) | 70.8 (65.9-75.3) | NS | 70.0 (62.6-79.5) | 72.6 (67.1-75.2) | NS |
| **Thrombodynamics** | | | | | | | |
| Tlag | min | 1.0 (0.9-1.3) | 1.1 (0.9-1.4) | NS | 1.3 (1.0-1.5) | 1.0 (0.8-1.2) | NS |
| Vi | µm/min | 59.8 (47.5-63.3) | 63.5 (55.6-67.6) | NS | 63.6 (42.9-68.8) | 62.1 (55.1-65.3) | NS |
| V | µm/min | 31.5 (25.3-33.8) | 34.9 (29.0-37.2) | NS | 33.9 (25.7-40.3) | 31.9 (28.7-36.2) | NS |
| CS | µm | 1248 (1046-1348) | 1280 (1114-1439) | NS | 1157 (824-1540) | 1280 (1136-1377) | NS |
| D | a.u. | 29650 (26619-31080) | 28458 (25256-30342) | NS | 22717 (19011-27637) | 29072 (26764-30546) | 0.037 |
| %Sp | n (%) | 1 (8.3) | 7 (29.2) | NS | 2 (22.2) | 6 (22.2) | NS |

IE – infectious endocarditis, EE – embolic events, APTT – activated partial thromboplastin time, FSC – forward lingt scattering, SSC – side light scattering, rest – resting platelets, act – activated platelets, NS – not significant

*For comparison of continuous data Mann-Whitney U-test was used. For comparison of categorical data Fisher’s exact test was used
