# Supplementary material for: Hypercoagulation detected by routine and global laboratory hemostasis assays in patients with infective endocarditis
Source: PLoS One. 2021 Dec 15;16(12):e0261429. doi: 10.1371/journal.pone.0261429 (PMC8673624; doi:10.1371/journal.pone.0261429)
Supplement: S4 Table — (DOCX) [file pone.0261429.s010.docx]

**S4 Table. Association of SNP data with outcomes.**

| SNP | Gene polymorphism |  | Genotype | All Patients (n=37) | IE with EE (n=13, 35.1%) | IE without EE (n=24, 64.9%) | p* | Fatal IE (n=9, 24.3%) | Non-fatal IE (n=28, 75.6%) | p* |
| --- | --- | --- | --- | --- | --- | --- | --- | --- | --- | --- |
|  |  |  |  | n (%all patients) | n (%measured) | n (%measured) |  | n (%measured) | n (%measured) |  |
| rs5918 | ITGB3 |  | T/T | 22 (59.5) | 6 (60.0) | 16 (69.6) | NS | 7 (100.0) | 15 (57.7) | NS |
|  |  |  | T/C | 10 (27.0) | 3 (30.0) | 7 (30.4) | NS | 0 (0.0) | 10 (38.5) | NA |
|  |  |  | C/C | 1 (2.7) | 1 (10.0) | 0 (0.0) | NS | 0 (0.0) | 1 (3.8) | NS |
|  |  |  | n/a | 4 (10.8) | 3 | 1 |  | 2 | 2 |  |
| rs1126643 | ITGA2 |  | C/C | 10 (27.0) | 1 (11.1) | 9 (37.5) | NS | 3 (42.9) | 7 (26.9) | NS |
|  |  |  | C/T | 13 (35.1) | 6 (66.7) | 7 (29.2) | NS | 2 (28.6) | 11 (42.3) | NS |
|  |  |  | T/T | 10 (27.0) | 2 (2) | 8 (33.3) | NS | 2 (28.6) | 8 (30.8) | NS |
|  |  |  | n/a | 4 (10.8) | 4 | 0 |  | 2 | 2 |  |
| rs6065 | GP1BA |  | C/C | 28 (75.7) | 8 (80.0) | 20 (87.0) | NS | 5 (62.5) | 23 (92.0) | NS |
|  |  |  | C/T | 5 (13.5) | 2 (20.0) | 3 (13.0) | NS | 3 (37.5) | 2 (8.0) | NS |
|  |  |  | T/T | 0 (0.0) | 0 (0.0) | 0 (0.0) | NS | 0 (0.0) | 0 (0.0) | NS |
|  |  |  | n/a | 4 (10.8) | 3 | 1 |  | 1 | 3 |  |
| rs1613662 | GP6 |  | T/T | 24 (64.9) | 8 (80.0) | 16 (66.7) | NS | 6 (75.0%) | 18 (69.2) | NS |
|  |  |  | T/C | 10 (27.0) | 2 (20.0) | 8 (33.3) | NS | 2 (25.0) | 8 (30.8) | NS |
|  |  |  | C/C | 0 (0.0) | 0 (0.0) | 0 (0.0) | NS | 0 (0.0) | 0 (0.0) | NS |
|  |  |  | n/a | 3 (8.1) | 3 | 0 |  | 1 | 2 |  |
| rs1799963 | G20210A FII |  | G/G | 32 (86.5) | 9 (100.0) | 23 (100.0) | NS | 7 (100.0) | 25 (100.0) | NS |
|  |  |  | G/A | 0 (0.0) | 0 (0.0) | 0 (0.0) | NS | 0 (0.0) | 0 (0.0) | NS |
|  |  |  | n/a | 5 (13.5) | 4 | 1 |  | 2 | 3 |  |
| rs6025 | FV Leiden |  | G/G | 29 (78.4) | 8 (100.0) | 21 (87.5) | NS | 7 (100.0) | 21 (87.5) | NS |
|  |  |  | G/A | 3 (8.1) | 0 (0.0) | 3 (12.5) | NS | 0 (0.0) | 3 (12.5) | NS |
|  |  |  | n/a | 5 (13.5) | 5 | 0 |  | 2 | 3 |  |
| rs1799 | SERPINE1 (PAI-1) |  | 4G/4G | 6 (16.2) | 0 (0.0) | 6 (46.1) | NS | 1 (33.3) | 5 (31.3) | NS |
|  |  |  | 5G/4G | 10 (27.0) | 5 (83.3) | 5 (38.5) | NS | 1 (33.3) | 9 (56.2) | NS |
|  |  |  | 5G/5G | 3 (8.1) | 1 (16.7) | 2 (15.4) | NS | 1 (33.3) | 2 (12.5) | NS |
|  |  |  | n/a | 18 (48.6) | 7 | 11 |  | 6 | 12 |  |
| rs5985 | F13 A1 |  | G/G | 21 (56.8) | 6 (60.0) | 15 (62.5) | NS | 4 (50.0) | 17 (65.4) | NS |
|  |  |  | G/T | 10 (27.0) | 3 (30.0) | 7 (29.2) | NS | 4 (50.0) | 6 (23.1) | NS |
|  |  |  | T/T | 3 (8.1) | 1 (10.0) | 2 (8.3) | NS | 0 (0.0) | 3 (11.5) | NS |
|  |  |  | n/a | 3 (8.1) | 3 | 0 |  | 1 | 2 |  |

*according to Fisher’s exact test. In the case of zero values in any of the groups, the reliability of difference was additionally verified by the χ^2^ Pearson test. NS – non-significant difference
